# Design and Validation of a Food Frequency Questionnaire to Evaluate the Consumption of Trans Fatty Acids in the Adult Population (FFQ-TFA)

**DOI:** 10.3390/ijerph192013097

**Published:** 2022-10-12

**Authors:** Edgar Ricardo Soto-Equihua, Claudia Ivonne Ramírez-Silva, Juana Elizabeth Elton-Puente, Jorge Luis Chávez-Servín, Pablo Gutiérrez-Lara, Elsa Fernanda Chávez-Alabat, María del Carmen Caamaño, Karina de la Torre-Carbot

**Affiliations:** 1 Facultad de Ciencias Naturales, Universidad Autónoma de Querétaro, Av. de las Ciencias S/N, Juriquilla, Querétaro 76320, Mexico; 2 Instituto Nacional de Salud Pública, Av. Universidad 655, Santa María Ahuacatitlán, Cuernavaca 62100, Mexico; 3 Facultad de Contaduría y Administración, Universidad Autónoma de Querétaro, Hidalgo S/N, Querétaro 76010, Mexico

**Keywords:** eating practices questionnaire (EPQ), food frequency questionnaire (FFQ), trans fatty acids (TFA), validation

## Abstract

Instruments for estimating the intake of food components can be useful in the prevention and/or treatment of diseases related to improper diet. There is, at present, no scientifically validated instrument for estimating consumption of trans fatty acids (TFA) in the Mexican population. The objective of this study was to design and validate such an instrument: a questionnaire that can be used to estimate consumption of TFA from food products. The questionnaire was applied to 162 students from the Autonomous University of Querétaro (UAQ). There were two phases to the study: (1) design of a food frequency questionnaire to assess consumption of trans fatty acids (FFQ-TFA) and an eating practices questionnaire (EPQ-TFA); (2) validation of the instrument. Content validity was measured by expert review and by Aiken’s V method, obtaining an overall score of 0.895. As final tests for the FFQ-TFA analysis, criterion validity was measured using Spearman’s correlation (r = 0.717, *p* < 0.01) and a linear regression (B = 0.668), considering the results of the 24-h dietary recall (24 HR); and reproducibility or temporal stability was measured using Pearson’s correlation (r = 0.406, *p* < 0.01). Subsequently, a Pearson correlation was applied between TFA consumption estimated by the FFQ-TFA-2 and the global score from the EPQ-TFA-2 (r = 0.351, *p* < 0.01). A Pearson correlation was applied between the EPQ-TFA-1 and the EPQ-TFA-2 (r = 0.575, *p* < 0.01). TFA consumption per day was 2.49 ± 1.32 g in the participating population, which was 1.04 ± 0.51% of their total kcal consumption.

## 1. Introduction

Improper eating habits and practices have been found to relate to many different health problems [1,2,3]. Among these habits and practices are excessive consumption of trans fatty acids (TFA) [1,4,5,6,7,8,9,10,11]. TFA have always been present in the human diet but recent years have seen a change in the foods from which these compounds are obtained and the amount consumed [12,13]. Foods closer to their natural origin contain much lower amounts of TFA than the highly processed food products that are more prevalent in modern diets. At present, approximately 1% to 5% of the TFA consumed are of natural origin. Beneficial health effects have been attributed to moderate consumption of such TFA. However, the remaining 95% to 99% are of industrial origin [5,7,14], and rising consumption of this type of TFA is considered one of the main causes of many of the health problems in the population today [7,15]. Industrialization in the second half of the 20th century brought new technologies for improving the characteristics of vegetable and animal oils used for commercial purposes. One of these technologies was hydrogenation, which transforms unsaturated fatty acids into saturated fatty acids [8,15], increasing shelf life and stability and reducing the cost of one raw material widely used by the food industry. At the same time, however, this process also results in the formation of TFA [1,4,7,8,15]. TFA can also result from the process of refining vegetable oils [8,12], as well as frying [12,15]. Elaidic acid (C18:1n9t) is the most abundant TFA of industrial origin in the human diet, accounting for 90%, followed by linoelaidic acid (C18:2n6t) [5,8,16].

The World Health Organization (WHO) and the Pan-American Health Organization (PAHO) both recommend eliminating TFA from the ideal diet, or at least limiting its consumption to less than 1% of total daily kilocalories, or 2 g/day [1,7,17]. Adhering to international warnings, the Mexican Ministry of Health recommends that TFA be limited to less than 1% of total daily calorie consumption [18].

According to many estimates, worldwide consumption of industrial TFA far exceeds these recommendations. Whether reported in grams or as a proportion of total calorie consumption, the existing estimates vary widely, primarily due to the different methodologies used [15]. Some studies report that worldwide consumption averages 1.4% of total kilocalories consumed (ranging from 0.2% to 6.5%), which would mean between 0.13 and 4.3 g per person per day [3,17,19,20,21]. In Mexico, there are three estimates on TFA consumption. In the first, using an FFQ and data obtained by the National Survey of Health and Nutrition in 2006 [20], consumption was estimated at 0.5 g/day, equivalent to 0.4% of the total daily consumption of kilocalories. The second estimate mentions an average consumption of 3.6% of total daily kilocalories [3]. The third [22] estimates TFA consumption by a small number of lactating women using a 24-h dietary recall (24 HR). In this study, the estimated daily consumption of TFA was 1.56 ± 0.75 g/day, or 0.66% ± 0.29% of total energy consumption.

In Mexico, there have been few studies to validate the use of the food frequency questionnaire (FFQ), especially in estimating consumption of a specific nutrient or dietary component [23], and no FFQ has been validated for assessing TFA consumption. Previous estimates of TFA consumption in Mexico have not been made systematically using tools previously validated or specific to TFA consumption. There are tools used in other countries but they cannot easily be applied in Mexico because of its distinct food products and patterns of consumption. The objective of this study was to design and validate a dietary questionnaire to assess the intake of TFA in Mexican adults.

## 2. Materials and Methods

**Figure 1:**
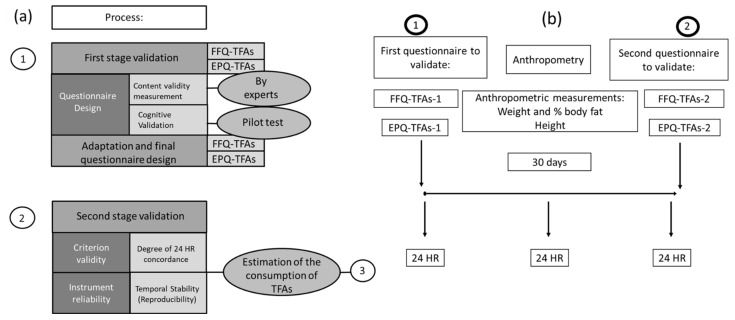
Stages of the study (**a**). Application of final questionnaires and 24 HR. Data collection and validation (**b**).

The study was carried out in two stages. During the first, the questionnaires were designed and content validity was measured by experts, while cognitive validation was measured during pilot testing. Based on the results, adjustments were made to arrive at the final questionnaire. During the second stage, criterion validity, specifically concurrent validity, was tested, and reliability testing of the instrument was also performed. The study yielded a set of estimates on TFA consumption in the participating population (Figure 1a).

This research was carried out according to the guidelines of the Declaration of Helsinki [24] and Mexico’s General Health Law on Health Research [25].

### 2.1. First Stage: Questionnaire Design and Cognitive Validation

Two questionnaires were designed: (1) Food Frequency Questionnaire (FFQ-TFA) and (2) Eating Practices Questionnaire (EPQ-TFA). Content validity and cognitive validation were carried out.

For content validity, a panel of seven experts was selected on the basis of their professional experience, specialty and academic degree [26] to evaluate the full questionnaire (FFQ-TFA and EPQ-TFA). Among the criteria examined were clarity of wording, clarity of instructions, internal coherence, adequate language, whether it measures what it intends, and the extent to which it fulfills its stated purpose. These experts also evaluated the logical distribution of the questions and the sufficiency of items to collect the desired information, and were asked for any other observations [27]. In the case of the FFQ-TFA, the appropriateness of the portion size was evaluated. Aiken’s V coefficient of validity was used [28,29], obtaining an overall value of 0.895 for both questionnaires: 0.90 for the FFQ-TFA and 0.89 for the EPQ-TFA [28]. According to this result, the experts found that the tool’s content was valid. Any observations made were incorporated, the necessary adjustments made, and questionnaires adapted in order to proceed with pilot testing and second-stage validation.

For pilot testing of the questionnaire, UAQ undergraduate students from various faculties (Environmental Geography, Veterinary and Zootechnics, Music, Anthropology, Law, Gastronomy and History) were invited to participate. The application of the instruments allowed the authors to verify the clarity and measure the application time of both questionnaires, with an average application time of 23 min [30,31]. Twenty-two questionnaires were applied, and further adjustments were made based on the results, which produced a final version of the questionnaire to be validated.

The FFQ-TFA designed were focused on the eating patterns of the Mexican population, based on the 24 HR of the National Health and Nutrition Survey 2012 and 2016. This study resulted in a total of 106 items, which were organized into 10 food groups: “Cereals,” “Milk and derivatives,” “Products of animal origin,” “*Pan dulce* and baked goods,” “Snacks,” “Prepared food,” “Fast food,” “Oils and fats,” “Sweet foods” and “Other preparations”. Response options regarding food consumption frequency were taken from Willet, 2012 [32]: “Never,” “Once a month,” “2–3 times per month,” “once a week,” “2–4 times per week,” “5–6 times per week,” and “every day.” A section was also included indicating how many times the food is consumed per day: “once a day,” “2–3 per day,” “4–5 per day,” and “6+ per day.” A section with the default portion size was included. In addition, the number of portions consumed was added: “1”, “2”, “3”, “4”, or “5”. To determine the content of TFA in foods, the “Tables of composition of fatty acids of common foods in the Mexican diet” were used [13]. When several brands of a single product exist, the products were grouped according to the type of food and a single item obtained based on the average of all the brands. For a better understanding of the items, a food atlas (visual aid) was made showing all the foods present in the designed FFQ-TFA.

The final EPQ-TFA consisted of 15 questions about dietary practices that relate to TFA consumption: type and frequency of food consumed outside the home, use of lipid sources for cooking (type of fats and oils), consumption of fried foods, interest in the nutritional content of the foods consumed, understanding of labeling, and consumption of some foods. The responses were multiple choice, and respondents could only select one answer to each. The responses were weighted to obtain a final score. Each question had a maximum score of 10 points, according to the frequency selected by the respondent, and the score assigned to each response varied depending on the number of options respondents could choose from in that question: 3.33, 6.66 and 10 for questions with 3 possible answers; 2.5, 5, 7.5 and 10 for questions with 4 possible answers; 2, 4, 6, 8 and 10 for 5 possible answers; 1.66, 3.32, 4.98, 6.64, 8.30 and 10 for the case of 6 possible answers; and 1.42, 2.84, 4.26, 5.68, 7.10, 8.52 and 10 for 7 possible answers. These values were assigned so that each question had the same maximum value or a proportional value according to the number of possible answers.

The FFQ-TFA and EPQ-TFA are shown in Supplementary File S1 and the food atlas in Supplementary File S2.

### 2.2. Second Stage: Criterion Validity and Instrument Reliability

Undergraduate students (*n* = 162) enrolled in the Faculty of Accounting and Administration (UAQ) with an average age of 21.5 ± 2.3 years were recruited. The students were given detailed information about the study, and those who agreed to participate in the study signed an informed consent statement. They attended a session where both questionnaires were applied for the first time (FFQ-TFA-1 and EPQ-TFA-1), in which they were shown a projection of the previously prepared image atlas. After 30 days, the two questionnaires were applied for a second time to the same group (FFQ-TFA-2 and EPQ-TFA-2), in order to measure reproducibility and temporal stability. During these same 30 days, the 24 HR data collection began using an adapted format [33] in order to determine habitual consumption: 2 during the week and 1 on the weekend (3-24 HR). During this same time, anthropometric measurements of height (SECA-213 stadiometer, Hamburg, Germany), weight and percentage of body fat (OMRON-HBF514 C bioimpedance scale, Kyoto, Japan) were taken. According to the procedures manual for nutrition projects, the body mass index (BMI) was obtained (Kg/m^2^) [33]. For its interpretation, the cutoff points established in NOM-043 were used [34] (Figure 1b).

A global dietary estimation was performed: the amount of carbohydrates, proteins and lipids consumed was estimated using the food composition tables [35]. The reported daily consumption of TFA was calculated in grams and as a percentage of the total kilocalories consumed, according to the average of the 3-24 HR analyzed during the study. TFA consumption was calculated by multiplying the frequency of consumption by the number of servings consumed per time by the grams of the serving, considering the TFA content in each food according to the fatty acid composition tables of frequent foods in the Mexican diet [13]. An Excel template was developed in which the foods considered were listed and the regular size of the portion of each food in grams was considered in the quantification according to the following formula:

$$\mathrm{TFAs}\;\left(g\right)=\overset{n}{\underset{1}{\sum }}\frac{v_{n}\times s_{n}\times g_{n}\times fa}{30}$$

where *v_n_* is the number of times the food was consumed in a month; *s_n_* is the number of servings consumed each time; *g_n_* is the grams of the standard portion; and *fa* is the mg of TFA that the food contains. The final data were divided by 30 to estimate the daily consumption of TFA consumed per day in grams.

To consider the content of TFA in oils or fats and powdered chicken bouillon used during the preparation of different dishes, a section called “Other preparations” was added to the FFQ-TFA, which lists dishes frequently consumed in the region, whose preparation regularly involves the use of oils or fats, and may or may not contain powdered chicken bouillon. The purpose of this section is—like the rest of the FFQ-TFA—to determine how many times a month each dish is consumed. To complete the analysis of TFA from oils and fats used in these preparations, another section called “Fat consumption” was added, which identifies the type of fat used for each dish, or if it is not used in that preparation. The resulting consumption of milliliters of oil or grams of fat was multiplied by its TFA content according to the Villalpando tables [13]. In another section called “seasoning consumption” the frequency with which the seasoning is used in each dish is identified. The grams of powdered chicken bouillon obtained were multiplied by the content of TFA contained in this product according to the same tables. Both data were added to the previously obtained data on TFA consumed per day in grams.

For the interpretation of the EPQ-TFA, a weighting was assigned to each of the responses to each question, with the maximum score per question being 10, according to the frequency selected by the respondent (which, in turn, indicated higher or more frequent TFA consumption), and a maximum score of 150 for the entire questionnaire. The value of each response option depended on the number of possible responses per question, ensuring that each question had the same total weight when the values were added up.

The criterion validity of the FFQ-TFA was verified by comparing TFA consumption (in grams) according to this questionnaire and TFA consumption (in grams) obtained using the reference method, the 24 HR, a tool which has been validated for dietary evaluation, and averaging the 3-24 HR results. To evaluate concordance, the Bland and Altman method was used [36,37,38,39,40].

The score for each of the questions on the questionnaire was obtained and then a final score was calculated; these were analyzed by means of a Pearson correlation with the TFA consumption obtained from the FFQ-TFA in order to identify whether the dietary practices included in the EPQ-TFA relate to the estimation of TFA consumption obtained by means of the FFQ-TFA.

To determine the reliability of the instrument, a temporal stability analysis was carried out using the test–retest method. The results of the FFQ-TFA-1 were compared with the FFQ-TFA-2, and the results of the EPQ-TFA-1 were compared with the EPQ-TFA-2.

### 2.3. Statistical Analysis

Descriptive analyses of the participating population were performed for age, weight, height, BMI, body fat, consumption of kilocalories, carbohydrates, proteins and lipids, as well as TFA consumption in grams and in proportion to total caloric content of the diet. A descriptive analysis of the most frequent eating practices that relate to TFA consumption according to the EPQ-TFA was carried out, in addition to the global score presented in means and standard deviations. Pearson correlations were calculated between TFA intake, BMI and percentage of body fat.

For the content validity of the FFQ-TFA and EPQ-TFA, Aiken’s V was used in a range of 0–1; where a result closer to 1 indicates a higher content validity. Values equal to or greater than 0.80 are considered acceptable [28,29].

To determine the strength of the association between the estimated consumption of the FFQ-TFA and the 24 HR, concurrent criterion validity was evaluated using Spearman’s correlation, since the 24 HR distributions were skewed to the right, along with a linear regression analysis. After performing the linear regression, the normality of residuals and the heteroscedasticity assumptions were tested, and a cross-validation was performed comparing the regression coefficient of 80% of the sample with that of the analysis considering the other 20%. This was carried out with the average daily consumption of TFA from 3-24 HR and the FFQ-TFA-2, since the latter measures 1 month in retrospect, the same period as when the 3-24 HR were applied; so, the results are comparable when measuring the same period of time [36,37,38].

The degree of concordance between the FFQ-TFA-2 and 3-24 HR and between the FFQ-TFA-1 and FFQ-TFA-2 was evaluated using the limits of agreement method developed by Bland and Altman, in order to compare intake between measurements made at different times and between two different instruments. The middle line of the graph usually generated during this test represents the mean of the differences, while the outer lines represent the limits of agreement ± standard deviations of the differences between FFQ-TFA-1 and FFQ-TFA-2, as well as between the FFQ-TFA and 24 HR methods [37,39,40].

The temporal stability or reproducibility between the two applications of the FFQ-TFA (FFQ-TFA-1 and FFQ-TFA-2) was assessed by applying a Pearson correlation coefficient between the consumption estimated in the first and the second questionnaires (test–retest). Temporal stability or reproducibility (test–retest) between the EPQ-TFA-1 and EPQ-TFA-2 was also evaluated.

A Pearson correlation was applied to study the relationship between TFA consumption determined by the FFQ-TFA and each of the items of the EPQ-TFA in both applications (FFQ-TFA-1 and EPQ-TFA-1; and FFQ-TFA- 2 and EPQ-TFA-2), as well as the relationship between TFA consumption determined by the FFQ-TFA-2 and the final score of the EPQ-TFA-2, the foregoing to identify which items relate to high consumption of TFA and determine whether there is a relationship between high TFA consumption estimated by the FFQ-TFA and a high final score.

All correlation analyses and linear regression analyses considered a 95% confidence interval and significance level *p* < 0.05.

## 3. Results and Discussions

The final version of the questionnaire was applied to 162 undergraduate students from UAQ’s Faculty of Accounting and Administration. The sample was made up of 108 women (67%) and 54 men (33%), with an average age of 21.5 ± 2.3 years. Of the total number of participants, 98.1% were single, 1.3% divorced, and only 0.6% married. In addition, 84.6% lived with their family, 4.3% with friends, 2.5% with their partner, and 8.6% alone.

The average weight of the participants was 60.70 ± 14.87 kg (78.65 ± 16.10 for men and 60.72 ± 14.87 for women), with a height of 163.48 ± 7.99 cm (171.7 ± 7.02 for men and 159.37 ± 4.54 for women), an average BMI of 24.77 ± 4.29 (26.58 ± 4.84 for men and 23.86 ± 3.68 for women) and a body fat percentage of 34.04 ± 8.17 (27.97 ± 8.05 for men and 37.08 ± 6.35 for women).

### 3.1. Validation of the FFQ-TFA and Estimation of TFA Consumption

#### 3.1.1. Criterion Validity

The consumption of energy, carbohydrates, proteins and lipids was estimated based on the 3-24 HR average (Table 1). TFA consumption/day was estimated using the FFQ-TFA-1, the FFQ-TFA-2 and the 3-24 HR (Table 2).

Estimated consumption values from the FFQ-TFA-1, the FFQ-TFA-2 and 3-24 HR were correlated, both in grams and as a percentage of kilocalories consumed in a day (Table 3).

**Figure 2:**
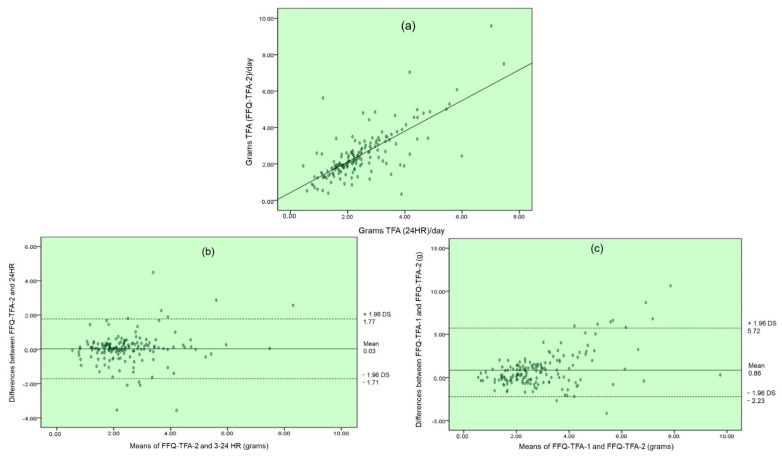
(**a**) Correlation between grams of TFA estimated by EPQ-TFA-2 and the average of the 3-24 HR. (**b**) Bland–Altman plot of the estimated differences in TFA consumption between FFQ-TFA-2 and 3-24 HR. (**c**) Bland–Altman plot of the estimated differences in TFA consumption between FFQ-TFA-1 and FFQ-TFA-2.

By correlating the estimated consumption from the FFQ-TFA-2 (2.49 ± 1.32) with the 3-24 HR average (2.46 ± 1.18), a value of r = 0.717 with a significance of *p* < 0.01 was obtained. This was the highest correlation and it coincides with what was expected, since the result of the FFQ-TFA-2 and the 3-24 HR (Figure 2a) pertain to the same time period. The FFQ-TFA-2 measures intake during the last month, the same period as when the 3-24 HR were taken, and shows estimated TFA consumption for that month. The results of this correlation (FFQ-TFA-2 with 3-24 HR) indicate that the two evaluation methods are comparable.

A linear regression was performed between the grams of TFA estimated from the FFQ-TFA-2 and the grams of TFA from the 3-24 HR average, obtaining a significant value of B = 0.668 and a confidence interval of 0.576–0.760. Heteroscedasticity of the model and normality of residuals were achieved. The results indicate a strong prediction of the 3-24 HR. Since the 95% confidence interval of the regression coefficient was less than 1, it may be inferred either that the FFQ-TFA overestimates TFA intake or the 3-24 HR underestimates TFA intake. However, further analysis indicates that, when considering the cutoff of 2 g, the small proportion of false positives and false negatives are the same (8%). The strong association between both measurements indicate an acceptable validity for estimating TFA (Table 4).

#### 3.1.2. Temporary Stability of FFQ-TFA

To assess temporal stability or reproducibility, a correlation was made between the TFA intake estimate obtained from the FFQ-TFA-1 and the FFQ-TFA-2, resulting in a correlation of r = 0.406 with a value for *p* <0.01, which is considered acceptable.

#### 3.1.3. Concordance Analysis

Concordance analysis was performed between FFQ-TFA-2 and 3-24 HR (Figure 2b) and between FFQ-TFA-1 and FFQ-TFA-2 (Figure 2c). A Bland–Altman plot was drawn of the results to allow for measurements located outside the limits of agreement. This concordance analysis provides a graphical assessment of the degree of agreement between the methods analyzed. Overall, 93.22% of the estimates (151 measurements) lie within the limits of agreement, while 3.70% (six measurements) are above the limits of agreement and 3.08% (five measurements) are below these limits. Most of the data are within the 95% confidence limit.

In the case of concordance between FFQ-TFA-1 and FFQ-TFA-2, 93.84% of the estimates (151 measurements) lie within the limits of agreement, while 4.93% (eight measurements) are above those limits and 1.23% (two measurements) are below them. Most of the data are within the 95% confidence limit.

#### 3.1.4. Estimated Consumption of TFA

TFA consumption/day was estimated using the FFQ-TFA1, the FFQ-TFA2 and the 3–42 HR (Table 2).

A significant difference was found between men and women in TFA consumption in grams using the 24 HR method (*p* = 0.043). This was not the case, however, when representing consumption in proportion to total kilocalories (Table 2).

Based on the results of both the FFQ-TFA-1 (3.36 ± 2.17 grams) (1.43 ± 0.99%) and FFQ-TFA-2 (2.49 ± 1.32 g) (1.04 ± 0.51%) and the 3-24 HR (2.46 ± 1.18 g) (1.02 ± 0.51), the intakes found in this research exceed 2 g/day and 1% of total kilocalories.

In Mexico, there are few estimates on TFA consumption. According to data obtained in 2006 by the National Survey of Health and Nutrition (and published in 2011) using a general FFQ not specific to TFA, a consumption of TFA of 0.5 g/day was reported, which represents 0.4% of total calorie consumption in a day. This questionnaire collected information from the 7 days prior to the survey, with a total of 101 foods divided into 12 groups, again, without being TFA-specific [20]. A second attempt at estimating TFA consumption in the Mexican population was made in 2014 by the Global Burden of Disease study, which reported TFA consumption amounting to 3.6% of the total energy consumed. This estimate was limited by the heterogeneity of the national databases used to obtain the data for each region [3]. Recently, consumption of TFA of industrial origin was estimated for a group of lactating women using 3-24 HR (two weekdays and one weekend), finding an estimated consumption of 1.66 ± 1.13 g/day or 0.70 ± 0.48% of total calories consumed [22]. The differences between these Mexican estimates of TFA consumption may be due to variations in the methods used but, above all, due to the use of tools not specifically developed for specifically estimating TFA intake. The estimates of this study show that consumption exceeds the national and international recommendation that TFA consumption be limited to no more than 1% of a person’s total daily kilocalorie intake [7,17,18]. This signals a need for strategies to reduce this type of TFA consumption in the population. Although the results of this study cannot reliably be extrapolated to the full population or the national level, they can be considered as a reference for the population studied.

Previous studies put average worldwide TFA consumption at the equivalent of 1.4%, with estimates ranging from 0.2% to 6.5% of total kilocalories (0.13 g to 4.3 g) [3,17,21]. The countries with the highest estimated TFA consumption values were: United States (7.8 g), Iceland (5.4 g), Austria (5 g), Chile (4.5 g) and Canada (3.4 g) [1,12,41]. Those where TFA values were similar to those found in this study are: United Kingdom (2.6 g), Germany, Peru and Puerto Rico (2 g), India (3 g) and Costa Rica (2.8 g) [12]. Other countries with lower TFA consumption are Korea and Japan (1.5 g), Italy and Greece (1.4 g), and the Netherlands (1.9 g) [1,12,41,42]. However, most countries exceed the WHO target for TFA of 2 g/day maximum or <1% of total Kcal. Denmark is a case that bears mention because government interventions succeeded in progressively lowering TFA intake by the population from 4.5 g/day in 1976 to 1.5 g/day in 1995 and, in 2004, succeeded in eliminating the intake of TFA from processed foods [1]. In some countries, TFA consumption has been estimated at less than 1%, including Barbados (0.2%), Haiti (0.4%), Ethiopia (0.6%) and Finland (0.8%) [3]. The differences in TFA consumption between countries may naturally be due to variations in eating patterns, habits and customs, access to foods with higher TFA content, as well as the amount of information available to consumers when choosing their foods. Of course, these differences may also arise because of variations in the estimation methods used [20].

In our study, estimated TFA was lower using the 3-24 HR method than by any of the FFQ-TFA: FFQ-TFA-1 (3.36 ± 2.17 g), FFQ-TFA-2 (2.49 ± 1.32 g), and the average of both questionnaires (2.92 ± 1.85 g), whereas the 3-24 HR estimate was 2.46 ± 1.18 g—a significant difference of *p* < 0.01 in all cases.

Some authors have found higher TFA values using FFQ than from other dietary estimation methods [36,43,44]. This is probably because data are drawn from a full month’s record of food consumed rather than for the one or several days covered by the 24 HR. In principle, this eliminates the risk of taking the measurement during an atypical day or two of diet and, because the FFQ is a tool capable of measuring food consumption retrospectively, the data should be more precise. Furthermore, our study’s accuracy is improved by its focus on foods with TFA content according to tables that specifically identify fatty acid composition. The specialization of this tool corrects for the risk of underestimation, which may explain why the TFA consumption estimates presented here are higher than those of previous studies.

### 3.2. Validation of the EPQ-TFA and its Analysis

#### 3.2.1. Relationship between TFA Consumption and Global EPQ-TFA Score

The maximum score of the EPQ-TFA is 150; the higher the score, the greater the consumption of TFA, which should, in principle, correlate with an estimate of greater consumption according to the FFQ-TFA.

A correlation was made between estimated TFA consumption in grams according to the FFQ-TGA-1 (3.36 ± 2.17) and the global EPQ-TFA-1 score (66.76 ± 16.75) in order to assess the relationship between high consumption and a high overall score. A value of r= 0.362 and a value for *p* of <0.01 were obtained. In terms of percentage of total Kcal consumption as well, a correlation was also found between the FFQ-TGA-1 estimation (1.43 ± 0.99) and the global EPQ-TFA-1 score, with r = 0.252 and *p* < 0.01, both indicating a significant positive correlation between the consumption estimation by the FFQ-TFA-1 and the global EPQ-TFA-1 score (Table 5).

The same correlation was made between the results of the second set of questionnaires. With a global score of 64.60 ± 16.31 in the second eating practices questionnaire (EPQ-TFA-2) and estimated TFA consumption in grams from the second food frequency questionnaire (FFQ-TFA-2) of 2.49 ± 1.32, the correlation showed a value of r= 0.351 and a *p*-value of <0.01. The FFQ-TFA-2 estimate of TFA intake in proportion to total Kcal consumption (1.04 ± 0.51) was also correlated with the EPQ-TFA-2 score, with r= 0.243 and *p* = 0.002. Again, this signals a significant positive correlation between FFQ-TFA-2 estimation and the global EPQ-TFA-2 score (Table 5): as estimated TFA consumption rises, so does the global EPQ-TFA-2 score.

Correlations were then drawn between each of the responses about eating practices on the EPQ-TFA and the TFA consumption estimation from the FFQ-TFA. The eating practices that related more closely to estimated TFA consumption were the frequency of consumption of fast food, cookies, *pan dulce* and fried snacks (Table 6), items which are regularly rich in TFA.

#### 3.2.2. Temporal Stability

To test for temporal stability, a correlation was made between the global scores obtained from the EPQ-TFA-1 (66.76 ± 16.75) and the EPQ-TFA-2 (64.60 ± 16.31). A correlation of r = 0.575 with a *p* value of <0.01 was found, indicating temporal stability between one measurement and another.

#### 3.2.3. Description of Food Practices

The results of both EPQ-TFA revealed that 22.8% to 30% of the population eats away from home three to four times a week. The basic presumption is that food away from home generally has a higher content of TFA. Between 28.4% and 32.1% of respondents say that the food they eat outside the home is prepared mainly through frying rather than through other methods involving less fat—boiling, baking or steaming, for example.

Over 90% of the population studied say they use primarily vegetable oil for cooking, and between 6.2% and 48.1% report reusing their cooking oil, a practice that increases the TFA content in food. Between 67.3% and 70.4% of the population avoids the consumption of fried and fatty foods sometimes, while only 15.4% to 16% of this population avoids it at all times. Eating more fried foods can translate to higher consumption of TFA. Interestingly, 48% of the respondents say they remove some of the visible fat from the food they eat.

Regarding labeling, only 6.2% to 8.6% of the population report always checking the labels of the products they consume, and only 3.7% to 6.8% of them say they can fully understand them. This reveals a problem, as most of the population does not take the precaution of checking food labels to verify their nutritional content or does not understand them, which can expose them to a higher TFA consumption.

The foods most frequently consumed by the population between meals were reported to be fruits or vegetables, French fries, churros and fried foods. Between 32.1% and 38.9% of the participants consume fast food one to two times per week—a class of food known to contain high amounts of TFA. Cookies are another of the foods rich in TFA, which, according to the EPQ-TFA, are consumed by 19.1% to 33.4% of the population between one or two times a week. Regarding the consumption of *pan dulce,* it was found that from 24.1% to 34% consume this one or two times a week. This is another food high in TFA content. Donuts are another of the foods included in the EPQ-TFA and, for these, consumption was somewhat lower in the population studied: between 33.4% and 37% consume them once a month. Asked about consumption of cake, from 42% to 49.4% of respondents say they consume cake once a month. This may be because cake is a food with a festive meaning and is, thus, consumed only sporadically, on special occasions. Finally, the EPQ-TFA revealed that between 27.8% and 35.8% of respondents consume fried snacks frequently—two to three times a month. It is important to study the consumption of these foods, since their intake is very popular among the population and they are also a significant source of TFA.

### 3.3. Relationship between TFA Consumption, other Consumption Variables, BMI and Percentage of Body Fat

A correlation was drawn between TFA consumption and other consumption variables, which indicated a positive and statistically significant relationship between TFA consumption in grams estimated by the 3-24 HR and the grams of carbohydrates consumed (r = 0.328 and *p* < 0.01). In addition, positive and statistically significant correlations were found between the consumption of lipids in grams and TFA consumption, estimated by both the FFQ-TFA-2 (r = 0.429 *p* < 0.01) and the 24 HR (r = 0.477 *p* < 0.01). Finally, a significant positive correlation was found between total kilocalorie intake and grams of TFA estimated by both the 24 HR (r = 0.435 *p* < 0.01) and the FFQ-TFA-2 (r = 0.376 *p* < 0.01). These results indicate a relationship between TFA consumption—estimated by either dietary method—and other consumption variables, such as carbohydrates, lipids and kilocalories. Not so with protein consumption. Therefore, it can be said that, in the population studied, the higher the consumption of lipids, carbohydrates and total kilocalories, the higher the consumption of TFA in the diet. Negative health effects may be caused both directly and indirectly by the consumption of these TFA, as it is related to other unhealthy eating habits and practices, such as the consumption of energy-dense foods rich in carbohydrates and lipids.

Despite the foregoing hypothesis, no significant correlation was found between TFA consumption and BMI among the subjects studied. Nor was any correlation found between TFA consumption and the percentage of body fat in this population. According to the results of our study, TFA consumption does correlate with the consumption of carbohydrates, lipids and total kilocalories. It might, thus, be supposed that TFA consumption would have some relationship to weight, BMI or percentage of body fat but no evidence of such a correlation was found in this study. Alternately, TFA consumption may be more clearly related to biochemical parameters and other factors at the cellular level, such as membrane damage, loss of fluidity, damage to cell signaling and an increase in proinflammatory cytokines, which can lead to cardiovascular problems [1,2,4,5,9,10]. Future research is required involving a greater number of anthropometric measurements and a broader sampling of populations with different BMI, including normal, overweight and obese subjects, to determine if there is a relationship between anthropometric characteristics and TFA consumption.

This work discusses the design and validation of a useful instrument for estimating the intake of TFA. The instrument may also be useful for analyzing some food practices that are related to the consumption of TFA. This questionnaire may furthermore be used in epidemiological studies because it can be applied quickly and at little cost, so that the estimates are generated from a consistent body of data rather than through diverse surveys using varied methodologies. Application of the FFQ-TFA designed in this study should allow for more precise, complete and replicable results. Further research would allow us to better understand TFA consumption and may be of use in relating TFA consumption with variables of interest involved in the development of certain chronic noncommunicable diseases. These, in turn, may be useful to policymakers and legislators at different levels in their efforts to reduce or eliminate TFA consumption in the population.

Estimates from studies on TFA consumption in other countries are always useful for comparative purposes. Direct comparisons are not always possible, however, because of differences in the methodologies used and in the expression of results. Although the WHO recommendation is given as a percentage of total kilocalories consumed, most scientific estimates are given in grams of TFA. Researchers should be encouraged to present results both in grams and as a percentage of total kilocalories consumed per day to facilitate international comparisons. Ongoing analysis of foods and products on the market is also necessary in order to keep food composition tables up to date, including information on the TFA content of the available products.

## 4. Conclusions

An instrument for measuring TFA intake was developed consisting of a Food Frequency Questionnaire focusing on foods containing trans fatty acids (FFQ-TFA), as well as an Eating Practices Questionnaire also targeting consumption of trans fatty acids (EPQ-TFA). For the FFQ-TFA, the level of criterion validity was found to be satisfactory, with a strong correlation between the FFQ-TFA and the 24 HR, which was used as the reference method; the proposed method showed temporal stability. It also showed satisfactory agreement between the two dietary analysis methods (FFQ-TFA and 3-24 HR) and between two successive applications (FFQ-TFA-1 and FFQ-TFA-2). The EPQ-TFA also presents temporary stability and was found useful in identifying food practices that relate to TFA consumption. As designed and validated, this instrument can be used to estimate TFA intake, both in epidemiological studies (because it can be quickly and cheaply applied) and in studies seeking to relate TFA consumption to other variables of interest. On a more basic level, TFA consumption found in the sample surveyed for this study exceeds the WHO recommendation, signaling the importance of programs to reduce or eliminate the presence of TFA in the diet.

## Figures and Tables

**Table 1:** Consumption of energy, carbohydrates, proteins and lipids (*n* = 162) according to 3-24 HR.

	Total	Women	Men	*p*
Energy (Kcal)	2184.30 ± 522.41	2060.45 ± 472.34	2432.01 ± 533.70	<0.01 **
Carbohydrates (g)	255.34 ± 72.62	241.36 ± 65.03	283.30 ± 79.29	<0.01 **
Carbohydrates (%)	46.76 ± 6.46	46.77 ± 6.13	46.73 ± 7.13	0.972
Proteins (g)	91.33 ± 25.57	86.30 ± 21.88	101.41 ± 29.39	<0.01 **
Proteins (%)	17.06 ± 4.00	17.02 ± 3.52	17.12 ± 4.86	0.896
Lipids (g)	87.76 ± 26.29	83.21 ± 24.21	96.85 ± 28.11	0.003 **
Lipids (%)	36.17 ± 5.68	36.19 ± 5.24	36.13 ± 6.53	0.955

Values are means ± standard deviation. 3-24 HR = the three 24-h dietary recall interviews conducted during questionnaire validation. g = grams. % = percentage. ** Significant difference according to Student’s *t*-test between men and women, *p* < 0.01.

**Table 2:** Estimated consumption of TFA/day (*n* = 162) by FFQ-TFA and 3-24 HR.

	Total		Women		Men			
	g	%	g	%	g	%	*p* (g)	*p* (%)
FFQ-TFA-1	3.36 ± 2.17	1.43 ± 0.99	3.21 ± 1.98	1.46 ± 1.03	3.65 ± 2.50	1.37 ± 0.91	0.264	0.551
FFQ-TFA-2	2.49 ± 1.32	1.04 ± 0.51	2.36 ± 1.20	1.05 ± 0.53	2.76 ± 1.51	1.02 ± 0.49	0.102	0.810
3-24 HR	2.46 ± 1.18	1.02 ± 0.51	2.31 ± 1.05	1.03 ± 0.55	2.75 ± 1.36	1.01 ± 0.41	0.043	0.829

Values are means ± standard deviation. FFQ-TFA-1 = Food Frequency questionnaire to estimate the consumption of trans fatty acids applied for the first time. FFQ-TFA-2 = Food Frequency questionnaire to estimate the consumption of trans fatty acids applied for the second time. 3-24 HR = the three 24-h dietary recall interviews conducted during questionnaire validation. g = grams. % = percentage of TFA consumption in relation to total daily Kcal according to the average of the 3-24 HR.

**Table 3:** Correlation between grams and percentages of TFA estimated by the 2 FFQ-TFA and the average of the 3-24 HR.

FFQ-TFA	3-24 H R	
	TFA (g) 3-24 HR (2.46 ± 1.18) ^b^	
	r ^a^	*p*
TFA (g) FFQ-TFA-1 (3.36 ± 2.17)	0.438	<0.01
TFA (g) FFQ-TFA-2 (2.49 ± 1.32)	0.717	<0.01
TFA (g) promedio FFQ-TFA-1 y FFQ-TFA-2 (2.92 ± 1.85)	0.604	<0.01
	TFA (%) 3-24 HR (1.02 ± 0.51) ^b^	
	r ^a^	*p*
TFA (%) FFQ-TFA-1 (1.43 ± 0.99)	0.403	<0.01
TFA (%) FFQ-TFA-2 (1.04 ± 0.51)	0.679	<0.01
TFA (%) Promedio FFQ-TFA-1 y FFQ-TFA-2 (1.24 ± 0.66%)	0.573	<0.01

^a^ Spearman’s rho coefficient; FFQ-TFA-1= Food Frequency questionnaire to estimate the consumption of trans fatty acids applied for the first time; FFQ-TFA-2 = Food Frequency questionnaire to estimate the consumption of trans fatty acids applied for the second time; 3-24 HR = the three 24-h dietary recall interviews conducted during questionnaire validation; g: grams; ^b^ such values are means ± standard deviation.

**Table 4:** Relationship between grams of TFA estimated by the FFQ-TFA-2 and the average of 3-24 HR.

	TFA (g) 3-24 HR (2.46 ± 1.18) ^b^		
	B ^a^	IC	*p*
TFA (g) FFQ-TFA-2 (2.49 ± 1.32)	0.668	(0.576–0.760)	<0.01

^a^ Linear regression; FFQ-TFA-2 = Food Frequency questionnaire to estimate the consumption of trans fatty acids applied for the second time; 3-24 HR = the three 24-h dietary recall interviews conducted during questionnaire validation; g: grams; B: Beta value; ^b^ values are means ± standard deviation.

**Table 5:** Relationship between the consumption of TFA estimated by the FFQ-TFA-2 and the global score of the EPQ-TFA-2.

	**TFA (g) FFQ-TFA-1 (3.36 ± 2.17) ^b^**		**% of Total Kcal FFQ-TFA-1 (1.43 ± 0.99) ^b^**	
	**R ^a^**	***p***	**R ^a^**	***p***
EPQ-TFA-1 score (66.76 ± 16.75) ^b^	0.362	<0.01	0.252	<0.01
	**TFA (g) FFQ-TFA-2 (2.49 ± 1.32)**		**% of Total Kcal FFQ-TFA-2 (1.04 ± 0.51)**	
	**R ^a^**	***p***	**R ^a^**	***p***
EPQ-TFA-2 score (64.60 ± 16.31)	0.351	<0.01	0.243	0.002

^a^ Pearson correlation; EPQ-TFA-1: Eating Practices questionnaire related to the consumption of trans fatty acids applied for the first time; FFQ-TFA-1 = Food Frequency questionnaire to estimate the consumption of trans fatty acids applied for the first time; EPQ-TFA-2: Eating Practices questionnaire related to the consumption of trans fatty acids applied for the second time; FFQ-TFA-2 = Food Frequency questionnaire to estimate the consumption of trans fatty acids applied for the second time; g: grams; % = percentage of total daily Kcal consumed in TFA according to the average of the 3-24 HR; ^b^ values are means ± standard deviation.

**Table 6:** Relationship of items of the EPQ-TFA-1 and EPQ-TFA-2 with the estimation of TFA consumption from the FFQ-TFA-1 and the FFQ-TFA-2.

Items	EPQ-TFA-1—FFQ-TFA-1		EPQ-TFA-2—FFQ-TFA-2.	
	Grams		Grams	
	r ^a^	*p*	r ^a^	*p*
How often do you eat out?	0.059	0.455	0.110	0.165
What method is used to prepare the food you usually eat outside the home?	0.110	0.165	0.173	0.028 *
What type of fat do you usually use to cook?	0.079	0.317	0.202	0.010 *
Do you reuse the oil for cooking?	0.027	0.733	−0.073	0.358
Do you avoid fried foods and fats, either at home or away from home?	0.188	0.017 *	0.148	0.060
How often do you check the labels of the foods you eat to check the fat content?	0.119	0.133	0.141	0.073
When you review the labels, do you fully understand them?	0.069	0.380	0.081	0.305
What do you do with the fat that can be seen with the naked eye in both liquid and solid foods?	0.116	0.140	−0.038	0.365
What foods do you usually eat between meals?	0.122	0.123	−0.136	0.085
How often do you eat fast food (pizza, hamburger, fried chicken, fried *quesadillas*, *sopes* or *gorditas*, etc.)?	0.288	<0.01 **	0.206	0.008 **
How often do you eat sweet cookies?	0.236	0.003 **	0.346	<0.01 **
How often do you eat *pan dulce*?	0.272	<0.01 **	0.290	<0.01 **
How often do you eat donuts?	0.377	<0.01 **	0.081	0.306
How often do you eat cakes?	0.308	<0.01 **	0.112	0.156
How often do you eat fried snacks (pork rinds, fried corn or industrialized fried foods)?	0.369	<0.01 **	0.378	<0.01 **

^a^ Pearson correlation; EPQ-TFA-1: Eating Practices questionnaire related to the consumption of trans fatty acids applied for the first time; EPQ-TFA-2: Eating Practices questionnaire related to the consumption of trans fatty acids applied for the second time; FFQ-TFA-2 = Food Frequency questionnaire to estimate the consumption of trans fatty acids applied for the second time; g: grams; * significance level < 0.05; ** significance level <0.01.

## Supplementary Materials

The following supporting information can be downloaded at: https://www.mdpi.com/article/10.3390/ijerph192013097/s1, Supplementary File S1. Food frequency and eating practices questionnaires to estimate the consumption of trans fatty acids (FFQ-TFA and EPQ-TFA); Cuestionarios de frecuencia alimentaria y prácticas alimentarias para estimar el consumo de ácidos grasos trans (FFQ-TFA y EPQ-TFA). Supplementary File S2. Food Atlas as visual aid with foods contained in the Food frequency questionnaire to estimate the consumption of trans fatty acids (Atlas de apoyo con imágenes de los alimentos contenidos en la Frecuencia de alimentos para estimar consumo de ácidos grasos trans).

## Abbreviations

(TFA)	Trans fatty acids
(FFQ)	Food frequency questionnaire
(FFQ-TFA)	Food frequency questionnaire to estimate the consumption of trans fatty acids
(FFQ-TFA-1)	Food frequency questionnaire to estimate the consumption of trans fatty acids, first application
(FFQ-TFA-2)	Food frequency questionnaire to estimate the consumption of trans fatty acids, second application
(EPQ)	Eating practices questionnaire
(EPQ-TFA)	Eating practices questionnaire related to the consumption of trans fatty acids
(EPQ-TFA-1)	Eating practices questionnaire related to the consumption of trans fatty acids, first application
(EPQ-TFA-2)	Eating practices questionnaire related to the consumption of trans fatty acids, second application
(UAQ)	Autonomous University of Querétaro
(WHO)	World Health Organization
(24 HR)	24-h dietary recall
(3-24 HR)	The three 24-h dietary recall interviews conducted during questionnaire validation
(BMI)	Body mass index
(PAHO)	Pan American Health Organization
