# Supplementary material for: Design and Validation of a Food Frequency Questionnaire to Evaluate the Consumption of Trans Fatty Acids in the Adult Population (FFQ-TFA)
Source: Int J Environ Res Public Health. 2022 Oct 12;19(20):13097. doi: 10.3390/ijerph192013097 (PMC9602579; doi:10.3390/ijerph192013097)
Supplement: Supplementary file 1 [file ijerph-19-13097-s001.zip › Supplementary File S1_English_2022_10_04.pdf]

Autonomous University of Queretaro.
Faculty of Natural Sciences.
Master in Integrative Clinical Nutrition.
Folio:____________________________

Supplementary File S1

Food frequency and eating practices questionnaires to estimate the consumption of trans fatty acids (FFQ-TFA and EPQ-TFA)

**Personal information**			
**1.1 Name:**____<br>Paternal surname Maternal surname First name (s)			
**1.2 Sex:** (____)<br>Female = 1<br>Male =2	**1.3 Age (years): (____)**	**1.4 Telephone number:**	**1.5 Date:**_____/_____/_____<br>Day /month/ year
**1.6 Undergraduate program: (____)**<br>Administration (1)<br>International Trade (2)<br>Accounting (3)	**1.7 Semester: (____)**	**1.8 Marital Status: (____)**<br>Single (1)<br>Married (2)<br>Divorced (3)<br>Widowed (4)	
**1.9 Lives with: (____)**<br>Parents or other family members (1)<br>Friends (2)<br>Partner (3)<br>Alone (4)		**1.12 BMI: (____)**	

**Weight:**	**1.10 Average**	**Height:**	**1.11 Average**	**1.13 % body fat**	**Average**
**1:**		**1:**		**1:**	
**2:**		**2:**		**2:**	

Autonomous University of Queretaro.
Faculty of Natural Sciences.
Master in Integrative Clinical Nutrition.
Folio:________________________

I. Food frequency questionnaire (FFQ-TFA).

PART 1

Instructions:

1.- Start by filling in the frequency section with the foods on the list that you ate in the last month.
2.- Do not leave any food blank. If you do not consume it, select the “never” box.
3.- Answer as precisely as possible, indicating both foods that you consume on their own and those that you add to other dishes.
4.- Choose one answer per group of columns. Mark with an X the box corresponding to the number of days per month or week on which you consume the food, then mark with another X the box corresponding to the times that you consume the food on those days.
5.- In the “number of portions consumed” column, mark with an X the number 1, 2, 3, 4 or 5 to indicate the number of portions you consume each time.
6.- To help you fill out the tables, you will be shown a series of images of the foods included.

On average, how often do you eat the following foods?

1.- Grains

		Days per month or week							Times a day				
Food	Portion	Never (0)	Once a month (1)	2-3 times per month (2)	Once a week (3)	2-4 times per week (4)	5-6 times per week (5)	Every day (6)	Once (7)	2-3 (8)	4-5 (9)	+6 t (10)	Number of portions consumed per sitting
1.1 Bolillo	1 medium piece (70 g)	0	1	2	3	4	5	6	7	8	9	10	1 I 2 I 3 I 4 I5
1.2 Sliced bread (type: Bimbo, Wonder, etc.)	1 slice (26 g)	0	1	2	3	4	5	6	7	8	9	10	1 I 2 I 3 I 4 I5
1.3 Toasted sliced bread (type: Bimbo, Wonder etc.)	1 slice (21 g)	0	1	2	3	4	5	6	7	8	9	10	1 I 2 I 3 I 4 I5
1.4 Cracker (type: saltine)	5 squares (16 g)	0	1	2	3	4	5	6	7	8	9	10	1 I 2 I 3 I 4 I5
1.5 Baguette	1/3 piece (70 g)	0	1	2	3	4	5	6	7	8	9	10	1 I 2 I 3 I 4 I5
1.6 Hamburger or sandwich bun	1 piece (60 g)	0	1	2	3	4	5	6	7	8	9	10	1 I 2 I 3 I 4 I5
1.7 Hot dog bun	1 piece (25 g)	0	1	2	3	4	5	6	7	8	9	10	1 I 2 I 3 I 4 I5
1.8 Wheat flour tortilla	3 pieces (75 g)	0	1	2	3	4	5	6	7	8	9	10	1 I 2 I 3 I 4 I5
1.9 Corn tortilla	3 pieces (100 g)	0	1	2	3	4	5	6	7	8	9	10	1 I 2 I 3 I 4 I5
1.10 Cooked rice	1 cup (50 g)	0	1	2	3	4	5	6	7	8	9	10	1 I 2 I 3 I 4 I5
1.11 Breakfast cereal (without milk)	½ cup (44 g)	0	1	2	3	4	5	6	7	8	9	10	1 I 2 I 3 I 4 I5

Autonomous University of Queretaro.
Faculty of Natural Sciences.
Master in Integrative Clinical Nutrition.
Folio:_______________________

Food	Portion	Never (0)	Once a month (1)	2-3 times per month (2)	Once a week (3)	2-4 times per week (4)	5-6 times per week (5)	Every day (6)	Once (7)	2-3 (8)	4-5 (9)	+6 t (10)	Number of portions consumed per sitting
**2.- Milk and derivatives**													
2.1 Whole milk	1 cup (216 g)	0	1	2	3	4	5	6	7	8	9	10	1 I 2 I 3 I 4 I5
2.2 Semi-skim milk	1 cup (247 g)	0	1	2	3	4	5	6	7	8	9	10	1 I 2 I 3 I 4 I5
2.3 Milk powder	2 heaping tablespoons (18 g)	0	1	2	3	4	5	6	7	8	9	10	1 I 2 I 3 I 4 I5
2.4 Evaporated milk (type: Carnation, Red Seal, Nestle)	1 cup (240 g)	0	1	2	3	4	5	6	7	8	9	10	1 I 2 I 3 I 4 I5
2.5 Plain yogurt or with fruit	¾ cup or individual package (156 g)	0	1	2	3	4	5	6	7	8	9	10	1 I 2 I 3 I 4 I5
2.6 Sour cream (type: Alpura, Lala, Great Value, etc.)	1 heaping tablespoon (15 g)	0	1	2	3	4	5	6	7	8	9	10	1 I 2 I 3 I 4 I5
2.7 Panela cheese	1 medium slice (30 g)	0	1	2	3	4	5	6	7	8	9	10	1 I 2 I 3 I 4 I5
2.8 Oaxaca cheese	1 medium slice (30 g)	0	1	2	3	4	5	6	7	8	9	10	1 I 2 I 3 I 4 I5
2.9 Manchego cheese	1 medium slice (30 g)	0	1	2	3	4	5	6	7	8	9	10	1 I 2 I 3 I 4 I5
2.10 Cream cheese	1 heaping tablespoon (15 g)	0	1	2	3	4	5	6	7	8	9	10	1 I 2 I 3 I 4 I5
2.11 Doble crema cheese	1 medium slice (30 g)	0	1	2	3	4	5	6	7	8	9	10	1 I 2 I 3 I 4 I5
**3.- Animal products**													
3.1 Beef	1 piece average steak (90 g)	0	1	2	3	4	5	6	7	8	9	10	1 I 2 I 3 I 4 I5
3.2 Pork	1 piece small loin or chop (60 g)	0	1	2	3	4	5	6	7	8	9	10	1 I 2 I 3 I 4 I5
3.3 Chicken (leg, thigh, breast)	1 medium piece (90 g)	0	1	2	3	4	5	6	7	8	9	10	1 I 2 I 3 I 4 I5
3.4 Egg (whole)	1 piece (62 g)	0	1	2	3	4	5	6	7	8	9	10	1 I 2 I 3 I 4 I5
3.5 Fresh fish	1 medium fillet (90g)	0	1	2	3	4	5	6	7	8	9	10	1 I 2 I 3 I 4 I5
3.6 Drained canned tuna	½ can (46 g)	0	1	2	3	4	5	6	7	8	9	10	1 I 2 I 3 I 4 I5
3.7 Sardine	1/3 can (70 g)	0	1	2	3	4	5	6	7	8	9	10	1 I 2 I 3 I 4 I5
3.8 Seafood (shrimp, octopus, etc.)	½ cup (60 g)	0	1	2	3	4	5	6	7	8	9	10	1 I 2 I 3 I 4 I5

Autonomous University of Queretaro.
Faculty of Natural Sciences.
Master in Integrative Clinical Nutrition.
Folio:____________________________

Food	Portion	Never (0)	Once a month (1)	2-3 times per month (2)	Once a week (3)	2-4 times per week (4)	5-6 times per week (5)	Every day (6)	Once (7)	2-3 (8)	4-5 (9)	+6 t (10)	Number of portions consumed per sitting
3.9 Sausage or hot dog	2 pieces (46 g)	0	1	2	3	4	5	6	7	8	9	10	1 I 2 I 3 I 4 I5
3.10 Ham	1 medium slice (16 g)	0	1	2	3	4	5	6	7	8	9	10	1 I 2 I 3 I 4 I5
3.11 Chorizo sausage	1 medium piece (41 g)	0	1	2	3	4	5	6	7	8	9	10	1 I 2 I 3 I 4 I5
3.12 Mortadella	1 medium slice (30 g)	0	1	2	3	4	5	6	7	8	9	10	1 I 2 I 3 I 4 I5
**4.- Pastry and bakery**													
4.1 Puff pastry horn	1 medium piece (70 g)	0	1	2	3	4	5	6	7	8	9	10	1 I 2 I 3 I 4 I5
4.2 Concha (Bakery)	1 medium piece (70 g)	0	1	2	3	4	5	6	7	8	9	10	1 I 2 I 3 I 4 I5
4.3 Concha (packaged)	1 piece (70 g)	0	1	2	3	4	5	6	7	8	9	10	1 I 2 I 3 I 4 I5
4.4 Bakery donut	1 piece (70 g)	0	1	2	3	4	5	6	7	8	9	10	1 I 2 I 3 I 4 I5
4.5 Packaged glazed donut	3 pieces (52 g)	0	1	2	3	4	5	6	7	8	9	10	1 I 2 I 3 I 4 I5
4.6 Packaged sugar donut	2 pieces (52 g)	0	1	2	3	4	5	6	7	8	9	10	1 I 2 I 3 I 4 I5
4.7 Bakery pancake	1 big slice (64 g)	0	1	2	3	4	5	6	7	8	9	10	1 I 2 I 3 I 4 I5
4.8 Packaged pancake	1 medium slice (32 g)	0	1	2	3	4	5	6	7	8	9	10	1 I 2 I 3 I 4 I5
4.9 Buñuelos (Fritters)	2 pieces (33 g)	0	1	2	3	4	5	6	7	8	9	10	1 I 2 I 3 I 4 I5
4.10 Banderilla bread	1 medium piece (80 g)	0	1	2	3	4	5	6	7	8	9	10	1 I 2 I 3 I 4 I5
4.11 Cookies without filling (type: oatmeal, amaranth, etc.)	2 pieces (40 g)	0	1	2	3	4	5	6	7	8	9	10	1 I 2 I 3 I 4 I5
4.12 Filled cookies (type: Emperor, Plativolos, Oreo, Prince, etc.)	5 pieces (57 g)	0	1	2	3	4	5	6	7	8	9	10	1 I 2 I 3 I 4 I5
4.13 Chocolate or butter cookies (type: Pastisetas)	4 pieces (30 g)	0	1	2	3	4	5	6	7	8	9	10	1 I 2 I 3 I 4 I5
4.14 Chocolate chip cookie (type: Chokis, Triki-trakes, Chips ahoy, etc.)	6 pieces (57 g)	0	1	2	3	4	5	6	7	8	9	10	1 I 2 I 3 I 4 I5
4.15 Wafer cookies (type: Soft creams, Cremax, etc.)	4 pieces (44 g)	0	1	2	3	4	5	6	7	8	9	10	1 I 2 I 3 I 4 I5
4.16 Fatty crackers (type: Crakets, Ritz, etc.)	12 pieces (35 g)	0	1	2	3	4	5	6	7	8	9	10	1 I 2 I 3 I 4 I5

Food	Portion	Never (0)	Once a month (1)	2-3 times per month (2)	Once a week (3)	2-4 times per week (4)	5-6 times per week (5)	Every day (6)	Once (7)	2-3 (8)	4-5 (9)	+6 t (10)	Number of portions consumed per sitting
4.17 Plain cookies (type: marías, little animals, etc.)	12 pieces (50 g)	0	1	2	3	4	5	6	7	8	9	10	1 I 2 I 3 I 4 I5
4.18 Shortbread cookie	4 pieces (60 g)	0	1	2	3	4	5	6	7	8	9	10	1 I 2 I 3 I 4 I5
4.1 Elephant ear (puff pastry)	1 medium piece (70 g)	0	1	2	3	4	5	6	7	8	9	10	1 I 2 I 3 I 4 I5
4.20 Fruit bars	2 medium pieces (55 g)	0	1	2	3	4	5	6	7	8	9	10	1 I 2 I 3 I 4 I5
4.21 Bakery cinnamon rolls	1 medium piece (70 g)	0	1	2	3	4	5	6	7	8	9	10	1 I 2 I 3 I 4 I5
4.22 Packaged cinnamon rolls	1 medium piece (70 g)	0	1	2	3	4	5	6	7	8	9	10	1 I 2 I 3 I 4 I5
4.23 Packaged muffin (type: Bimbo, Tía Rosa, etc.)	2 medium pieces (60 g)	0	1	2	3	4	5	6	7	8	9	10	1 I 2 I 3 I 4 I5
4.24 Tiramisu	½ piece (41 g)	0	1	2	3	4	5	6	7	8	9	10	1 I 2 I 3 I 4 I5
4.25 Chocolate or carrot cake	1 big slice (163 g)	0	1	2	3	4	5	6	7	8	9	10	1 I 2 I 3 I 4 I5
4.26 Packaged snack cake (type: Gansito, Penguin, Negrito, Submarine, Choco Roles, etc.)	1 piece (59 g)	0	1	2	3	4	5	6	7	8	9	10	1 I 2 I 3 I 4 I5
4.27 Cereal bar (type: Special K, All bran, Bran frut, etc.)	1 small piece (25 g)	0	1	2	3	4	5	6	7	8	9	10	1 I 2 I 3 I 4 I5
**5.- Snack**													
5.1 Corn chips (type: Fritos, Takis, Churrumais, Runners, etc.)	1 medium package (46 g)	0	1	2	3	4	5	6	7	8	9	10	1 I 2 I 3 I 4 I5
5.2 Potato chips (type: Sabritas, Chips, Pringles, Ruffles, etc.)	1 medium package (45 g)	0	1	2	3	4	5	6	7	8	9	10	1 I 2 I 3 I 4 I5
5.3 Microwave popcorn	½ package (30 g)	0	1	2	3	4	5	6	7	8	9	10	1 I 2 I 3 I 4 I5
5.4 Packaged fried plantain chips	1 medium package (32 g)	0	1	2	3	4	5	6	7	8	9	10	1 I 2 I 3 I 4 I5

Autonomous University of Queretaro.
Faculty of Natural Sciences.
Master in Integrative Clinical Nutrition.
Folio:___________________________

Food	Portion	Never (0)	Once a month (1)	2-3 times per month (2)	Once a week (3)	2-4 times per week (4)	5-6 times per week (5)	Every day (6)	Once (7)	2-3 (8)	4-5 (9)	+6 t (10)	Number of portions consumed per sitting
**6.- Prepared food**													
6.1 Mexican snacks: sopes, quesadillas, gorditas, fried tacos.	2 pieces (144 g)	0	1	2	3	4	5	6	7	8	9	10	1 I 2 I 3 I 4 I5
6.2 Enchiladas	3 pieces (150 g)	0	1	2	3	4	5	6	7	8	9	10	1 I 2 I 3 I 4 I5
6.3 Barbecue taco	1 piece (70 g)	0	1	2	3	4	5	6	7	8	9	10	1 I 2 I 3 I 4 I5
6.4 Tamale	1 piece (200 g)	0	1	2	3	4	5	6	7	8	9	10	1 I 2 I 3 I 4 I5
6.5 Home fries	1 cup (75 g)	0	1	2	3	4	5	6	7	8	9	10	1 I 2 I 3 I 4 I5
6.6 Fried flour cracklings	1 bag (18 g)	0	1	2	3	4	5	6	7	8	9	10	1 I 2 I 3 I 4 I5
6.7 Torta	1 medium piece (150 g)	0	1	2	3	4	5	6	7	8	9	10	1 I 2 I 3 I 4 I5
6.8 Empanada	1 piece (120 g)	0	1	2	3	4	5	6	7	8	9	10	1 I 2 I 3 I 4 I5
6.9 Hotcake	2 medium pieces (120 g)	0	1	2	3	4	5	6	7	8	9	10	1 I 2 I 3 I 4 I5
6.10 Churro with sugar	1 piece (100 g)	0	1	2	3	4	5	6	7	8	9	10	1 I 2 I 3 I 4 I5
**7.- Fast food**													
7.1 French fries	1 package (53 g)	0	1	2	3	4	5	6	7	8	9	10	1 I 2 I 3 I 4 I5
7.2 Fried chicken (type: KFC)	2 medium pieces (108 g)	0	1	2	3	4	5	6	7	8	9	10	1 I 2 I 3 I 4 I5
7.3 Beef Burger	1 medium piece (160 g)	0	1	2	3	4	5	6	7	8	9	10	1 I 2 I 3 I 4 I5
7.4 Chicken Burger	1 medium piece (160 g)	0	1	2	3	4	5	6	7	8	9	10	1 I 2 I 3 I 4 I5
7.5 Pizza	1 medium slice (135 g)	0	1	2	3	4	5	6	7	8	9	10	1 I 2 I 3 I 4 I5
7.6 Burrito	1 medium piece (120 g)	0	1	2	3	4	5	6	7	8	9	10	1 I 2 I 3 I 4 I5
7.7 Corn dog	1 piece (120 g)	0	1	2	3	4	5	6	7	8	9	10	1 I 2 I 3 I 4 I5
**8.- Oils and fats**													
8.1 Mayonnaise	1 heaping tablespoon (9 g)	0	1	2	3	4	5	6	7	8	9	10	1 I 2 I 3 I 4 I5
8.2 Butter	1 teaspoon (5 g)	0	1	2	3	4	5	6	7	8	9	10	1 I 2 I 3 I 4 I5
8.3 Margarine	1 teaspoon (5 g)	0	1	2	3	4	5	6	7	8	9	10	1 I 2 I 3 I 4 I5

Food	Portion	Never (0)	Once a month (1)	2-3 times per month (2)	Once a week (3)	2-4 times per week (4)	5-6 times per week (5)	Every day (6)	Once (7)	2-3 (8)	4-5 (9)	+6 t (10)	Number of portions consumed per sitting
8.4 Peanut butter	1 heaping tablespoon (27 g)	0	1	2	3	4	5	6	7	8	9	10	1 I 2 I 3 I 4 I5
8.5 Olive oil	1 tablespoon (10 g)	0	1	2	3	4	5	6	7	8	9	10	1 I 2 I 3 I 4 I5
8.6 Peanuts and pumpkin seeds	½ package (35 g)	0	1	2	3	4	5	6	7	8	9	10	1 I 2 I 3 I 4 I5
8.7 Avocado	1/3 piece (37 g)	0	1	2	3	4	5	6	7	8	9	10	1 I 2 I 3 I 4 I5
9.- Sweet foods													
9.1 Chocolate bar (type: Carlos V, Tin Larín, Vaquita, Hershey's, etc.)	1 small bar (23 g)	0	1	2	3	4	5	6	7	8	9	10	1 I 2 I 3 I 4 I5
9.2 Chocolate with biscuit (type: Tin Larín, Bocadín, Kit-kat, etc.)	1 medium piece (33 g)	0	1	2	3	4	5	6	7	8	9	10	1 I 2 I 3 I 4 I5
9.3 Candied chocolate (type: M&M, lunettes, etc.)	1 small package (24 g)	0	1	2	3	4	5	6	7	8	9	10	1 I 2 I 3 I 4 I5
9.4 Gumdrops	2 small pieces (7 g)	0	1	2	3	4	5	6	7	8	9	10	1 I 2 I 3 I 4 I5
9.5 Condensed milk	1 tablespoon (19 g)	0	1	2	3	4	5	6	7	8	9	10	1 I 2 I 3 I 4 I5

Autonomous University of Queretaro.
Faculty of Natural Sciences.
Master in Integrative Clinical Nutrition.
Folio:________________________

PART 2. FAT CONSUMPTION

<table>
<tr><td></td><td colspan="8">What type of fat do you use to prepare the following dishes?

FOR EACH DISH, MARK "1" IN THE TYPE OF FAT OR OIL YOU USUALLY USE ,AND "0" IN THE TYPE OF FAT OR OIL YOU DO NOT USE</td><td>Usually, how do you prepare these foods.
Roasted (1)
Stewed (2)
Fried (3)</td></tr>
<tr><td><u>Dish</u></td><td>Vegetable oil (1)</td><td>Lard (2)</td><td>Vegetable shortening (3)</td><td>Margarine (4)</td><td>Butter (5)</td><td>Spray oil (6)</td><td>I don't use fats or oils (7)</td><td>I do not consume (8)</td><td></td></tr>
<tr><td>2.1 Rice</td><td>|___|</td><td>|___|</td><td>|___|</td><td>|___|</td><td>|___|</td><td>|___|</td><td>|___|</td><td>|___|</td><td>|___|</td></tr>
<tr><td>2.2 Fried noodle soup</td><td>|___|</td><td>|___|</td><td>|___|</td><td>|___|</td><td>|___|</td><td>|___|</td><td>|___|</td><td>|___|</td><td>|___|</td></tr>
<tr><td>2.3 Beans</td><td>|___|</td><td>|___|</td><td>|___|</td><td>|___|</td><td>|___|</td><td>|___|</td><td>|___|</td><td>|___|</td><td>|___|</td></tr>
<tr><td>2.4 Refried beans</td><td>|___|</td><td>|___|</td><td>|___|</td><td>|___|</td><td>|___|</td><td>|___|</td><td>|___|</td><td>|___|</td><td>|___|</td></tr>
<tr><td>2.5 Fried bananas</td><td>|___|</td><td>|___|</td><td>|___|</td><td>|___|</td><td>|___|</td><td>|___|</td><td>|___|</td><td>|___|</td><td>|___|</td></tr>
<tr><td>2.6 Fried eggs</td><td>|___|</td><td>|___|</td><td>|___|</td><td>|___|</td><td>|___|</td><td>|___|</td><td>|___|</td><td>|___|</td><td>|___|</td></tr>
<tr><td>2.7 Scrambled eggs</td><td>|___|</td><td>|___|</td><td>|___|</td><td>|___|</td><td>|___|</td><td>|___|</td><td>|___|</td><td>|___|</td><td>|___|</td></tr>
<tr><td>2.8 Chicken</td><td>|___|</td><td>|___|</td><td>|___|</td><td>|___|</td><td>|___|</td><td>|___|</td><td>|___|</td><td>|___|</td><td>|___|</td></tr>
<tr><td>2.9 Beef</td><td>|___|</td><td>|___|</td><td>|___|</td><td>|___|</td><td>|___|</td><td>|___|</td><td>|___|</td><td>|___|</td><td>|___|</td></tr>
<tr><td>2.10 Fish</td><td>|___|</td><td>|___|</td><td>|___|</td><td>|___|</td><td>|___|</td><td>|___|</td><td>|___|</td><td>|___|</td><td>|___|</td></tr>
<tr><td>2.11 Battered vegetables</td><td>|___|</td><td>|___|</td><td>|___|</td><td>|___|</td><td>|___|</td><td>|___|</td><td>|___|</td><td>|___|</td><td>|___|</td></tr>
<tr><td>2.12 Potato pancakes or fries</td><td>|___|</td><td>|___|</td><td>|___|</td><td>|___|</td><td>|___|</td><td>|___|</td><td>|___|</td><td>|___|</td><td>|___|</td></tr>
<tr><td>2.13 Battered meat or chicken patties</td><td>|___|</td><td>|___|</td><td>|___|</td><td>|___|</td><td>|___|</td><td>|___|</td><td>|___|</td><td>|___|</td><td>|___|</td></tr>
<tr><td>2.14 Breaded meat or chicken</td><td>|___|</td><td>|___|</td><td>|___|</td><td>|___|</td><td>|___|</td><td>|___|</td><td>|___|</td><td>|___|</td><td>|___|</td></tr>
<tr><td>2.15 Mexican snacks (quesadillas, tacos, flautas, gorditas, sopes, tamales)</td><td>|___|</td><td>|___|</td><td>|___|</td><td>|___|</td><td>|___|</td><td>|___|</td><td>|___|</td><td>|___|</td><td>|___|</td></tr>
</table>

Autonomous University of Queretaro.
Faculty of Natural Sciences.
Master in Integrative Clinical Nutrition.
Folio:_______________________

*NOTE: IF YOU USE MORE THAN ONE TYPE OF FAT IN EACH FOOD PREPARATION, MARK THE CORRESPONDING BOXES

CONSUMPTION OF POWDERED CHICKEN BOUILLON

	**How often do you use powdered chicken bouillon to prepare the following dishes?** FOR EACH DISH, MARK WITH “1” ACCORDING TO THE FREQUENCY WITH WHICH YOU USE IT. IF YOU DO NOT CONSUME THIS DISH, MARK THE OPTION "Does not apply"						
**Food preparations**	Does not apply	I do not use it	I use little (less than Once per week)	I use a few times a week (2-3 times)	I frequently use (4-6 times)	I use every day	I don’t know
3.1 Rice	|___|	|___|	|___|	|___|	|___|	|___|	|___|
3.2 Fried noodle soup	|___|	|___|	|___|	|___|	|___|	|___|	|___|
3.3 Beans	|___|	|___|	|___|	|___|	|___|	|___|	|___|
3.4 Refried beans	|___|	|___|	|___|	|___|	|___|	|___|	|___|
3.5 Fried bananas	|___|	|___|	|___|	|___|	|___|	|___|	|___|
3.6 Fried eggs	|___|	|___|	|___|	|___|	|___|	|___|	|___|
3.7 Scrambled eggs	|___|	|___|	|___|	|___|	|___|	|___|	|___|
3.8 Chicken	|___|	|___|	|___|	|___|	|___|	|___|	|___|
3.9 Beef	|___|	|___|	|___|	|___|	|___|	|___|	|___|
3.10 Fish	|___|	|___|	|___|	|___|	|___|	|___|	|___|
3.11 Battered vegetables	|___|	|___|	|___|	|___|	|___|	|___|	|___|
3.12 Potato pancakes or fries	|___|	|___|	|___|	|___|	|___|	|___|	|___|
3.13 Battered meat or chicken patties	|___|	|___|	|___|	|___|	|___|	|___|	|___|
3.14 Breaded meat or chicken	|___|	|___|	|___|	|___|	|___|	|___|	|___|
3.15 Mexican snacks (quesadillas, tacos, flautas, gorditas, sopes, tamales)	|___|	|___|	|___|	|___|	|___|	|___|	|___|

Autonomous University of Queretaro.
Faculty of Natural Sciences.
Master in Integrative Clinical Nutrition.
Folio:________________________

II. Eating practices questionnaire related to the consumption of trans fatty acids (EPQ-TFA)

Instructions: Choose one answer option for each question, unless the question indicates that multiple options can be chosen. Circle the answer that best answers the question according to the activities you do.

1. How often do you eat out?
   a. Every day (5)
   b. 5-6 times a week (4)
   c. 3-4 times a week (3)
   d. 1-2 times a week (2)
   e. 1-2 times a month (1)
   f. Never (0)

2. What method is used to prepare the food you usually eat outside the home?
   a. Fried (4)
   b. Stewed (3)
   c. Roasted (2)
   d. Baked or grilled (1)
   e. Boiled (0)
   f. Steamed (0)

3. What type of fat do you usually use to cook?
   a. Margarine (5)
   b. Vegetable shortening (4)
   c. Lard (3)
   d. Butter (2)
   e. Vegetable oil (1)

4. Do you reuse the oil for cooking?
   a. Always (2)
   b. Sometimes (1)
   c. Never (0)

5. Do you avoid fried foods and fats, either at home or away from home?
    a. Always (2)
    b. Sometimes (1)
    c. Never (0)

6. How often do you check the labels of the foods you eat to check the fat content?
    a. Never (3)
    b. Sometimes (2)
    c. Frequently (1)
    d. Always (0)

7. When you review the labels, do you fully understand them?
    a. Never (3)
    b. Sometimes (2)
    c. Frequently (1)
    d. Always (0)

8. What do you do with the fat that can be seen with the naked eye in both liquid and solid foods?
    a. I eat it (2)
    b. I remove a little of it (1)
    c. I take it all off (0)

9. What foods do you usually eat between meals?
    a. Cookies or sweet bread (3)
    b. French fries, churros or chips (2)
    c. Peanuts or seeds (1)
    d. Candy (0)
    e. Fruits or vegetables (0)
    f. None of the above (0)

10. How often do you eat fast food? (pizza, hamburger, fried chicken, fried quesadillas, sopes, gorditas etc.)
    a. Every day (6)
    b. 5-6 times a week (5)

c. 3-4 times a week (4)
d. 1-2 times a week (3)
e. 2-3 times a month (2)
f. Once a month (1)
g. Never (0)

11. How often do you eat sweet cookies?
    a. Every day (6)
    b. 5-6 times a week (5)
    c. 3-4 times a week (4)
    d. 1-2 times a week (3)
    e. 2-3 times a month (2)
    f. Once a month (1)
    g. Never (0)

12. How often do you eat *pan dulce*?
    a. Every day (6)
    b. 5-6 times a week (5)
    c. 3-4 times a week (4)
    d. 1-2 times a week (3)
    e. 2-3 times a month (2)
    f. Once a month (1)
    g. Never (0)

13. How often do you eat donuts?
    a. Every day (6)
    b. 5-6 times a week (5)
    c. 3-4 times a week (4)
    d. 1-2 times a week (3)
    e. 2-3 times a month (2)
    f. Once a month (1)
    g. Never (0)
    h.

14. How often do you eat cake?
    a. Every day (6)
    b. 5-6 times a week (5)
    c. 3-4 times a week (4)
    d. 1-2 times a week (3)
    e. 2-3 times a month (2)
    f. Once a month (1)
    g. Never (0)

15. How often do you eat fried snacks (pork rinds, fried corn or industrialized fried foods)?
    a. Every day (6)
    b. 5-6 times a week (5)
    c. 3-4 times a week (4)
    d. 1-2 times a week (3)
    e. 2-3 times a month (2)
    f. Once a month (1)
    g. Never (0)

Total points:___________________________

II. Eating practices questionnaire related to the consumption of trans fatty acids (EPQ-TFA)

Instructions: For Interviewer Use Only

1. How often do you eat out?
   a. Every day (5)
   b. 5-6 times a week (4)
   c. 3-4 times a week (3)
   d. 1-2 times a week (2)
   e. 1-2 times a month (1)
   f. Never (0)

a = 10
b = 8.3
c = 6.64
d = 4.98
e = 3.32
f = 1.66

2. What method is used to prepare the food you usually eat outside the home?
   a. Fried (4)
   b. Stewed (3)
   c. Roasted (2)
   d. Baked or grilled (1)
   e. Boiled (0)
   f. Steamed (0)

a = 10
b = 8.3
c = 6.64
d = 4.98
e = 3.32
f = 1.66

3. What type of fat do you usually use to cook?
   a. Margarine (5)
   b. Vegetable shortening (4)
   c. Lard (3)
   d. Butter (2)
   e. Vegetable oil (1)

a = 10
b = 8
c = 6
d = 4
e = 2

4. Do you reuse the oil for cooking?
   a. Always (2)
   b. Sometimes (1)
   c. Never (0)

a = 10
b = 6.66
c = 3.33

5. Do you avoid fried foods and fats, either at home or away from home?
    a. Always (2)
    b. Sometimes (1)
    c. Never (0)

a = 10
b = 6.66
c = 3.33

6. How often do you check the labels of the foods you eat to check the fat content?
    a. Never (3)
    b. Sometimes (2)
    c. Frequently (1)
    d. Always (0)

a = 10
b = 7.5
c = 5
d = 2.5

7. When you review the labels, do you fully understand them?
    a. Never (3)
    b. Sometimes (2)
    c. Frequently (1)
    d. Always (0)

a = 10
b = 7.5
c = 5
d = 2.5

8. What do you do with the fat that can be seen with the naked eye in both liquid and solid foods?
    a. I eat it (2)
    b. I remove a little of it (1)
    c. I take it all off (0)

a = 10
b = 6.66
c = 3.33

9. What foods do you usually eat between meals?
    a. Cookies or sweet bread (3)
    b. French fries, churros or chips (2)
    c. Peanuts or seeds (1)
    d. Candy (0)
    e. Fruits or vegetables (0)
    f. None of the above (0)

a = 10
b = 8.52
c = 7.1
d = 5.68
e = 4.26
f = 2.84

10. How often do you eat fast food? (pizza, hamburger, fried chicken, fried quesadillas, sopes, gorditas etc.)
    a. Every day (6)
    b. 5-6 times a week (5)
    c. 3-4 times a week (4)
    d. 1-2 times a week (3)
    e. 2-3 times a month (2)
    f. Once a month (1)
    g. Never (0)

a = 10
b = 8.52
c = 7.1
d = 5.68
e = 4.26
f = 2.84
g =1.42

11. How often do you eat sweet cookies?
    a. Every day (6)
    b. 5-6 times a week (5)
    c. 3-4 times a week (4)
    d. 1-2 times a week (3)
    e. 2-3 times a month (2)
    f. Once a month (1)
    g. Never (0)

a = 10
b = 8.52
c = 7.1
d = 5.68
e = 4.26
f = 2.84
g =1.42

12. How often do you eat *pan dulce*?
    a. Every day (6)
    b. 5-6 times a week (5)
    c. 3-4 times a week (4)
    d. 1-2 times a week (3)
    e. 2-3 times a month (2)
    f. Once a month (1)
    g. Never (0)

a = 10
b = 8.52
c = 7.1
d = 5.68
e = 4.26
f = 2.84
g =1.42

13. How often do you eat donuts?
    a. Every day (6)
    b. 5-6 times a week (5)
    c. 3-4 times a week (4)
    d. 1-2 times a week (3)
    e. 2-3 times a month (2)
    f. Once a month (1)
    g. Never (0)

a = 10
b = 8.52
c = 7.1
d = 5.68
e = 4.26
f = 2.84
g =1.42

14. How often do you eat cake?
    a. Every day (6)
    b. 5-6 times a week (5)
    c. 3-4 times a week (4)
    d. 1-2 times a week (3)
    e. 2-3 times a month (2)
    f. Once a month (1)
    g. Never (0)

a = 10
b = 8.52
c = 7.1
d = 5.68
e = 4.26
f = 2.84
g =1.42

15. How often do you eat fried snacks (pork rinds, fried corn or industrialized fried foods)?
    a. Every day (6)
    b. 5-6 times a week (5)
    c. 3-4 times a week (4)
    d. 1-2 times a week (3)
    e. 2-3 times a month (2)
    f. Once a month (1)
    g. Never (0)

a = 10
b = 8.52
c = 7.1
d = 5.68
e = 4.26
f = 2.84
g = 1.42

g =1.42

Total points:___________________________

Mexican foods mentioned in the questionnaires:

- Banderilla: A stick of puff pastry glazed with caramelized sugar.
- Bolillo: A large baked roll similar to a baguette but shorter in length.
- Buñuelo: a thin, round, fried pastry, often dusted with cinnamon sugar
- Burrito: a Mexican dish consisting of a flour tortilla wrapped around a filling of meat, cheese, fried beans, etc.
- Churros: sticks of extruded fried dough rolled in sugar
- Concha: A soft variety of pan dulce topped with a crust made of shortening and sugar
- Doble crema cheese: an artisanal fresh cheese, high in humidity and fat content
- Enchiladas: Filled soft tacos smothered in sauce
- Flautas: Fried rolled corn tortilla filled with chicken
- Gorditas: A fried corn masa patty stuff with cheese or other savory fillings.
- Oaxaca cheese: A medium-aged cheese formed into knots, similar to cured mozzarella or string cheese
- Pan dulce: Any one of a variety of sweet baked goods or pastries
- Panela cheese: A smooth fresh cheese similar to fresh mozzarella
- Quesadilla: a toasted tortilla filled with cheese and sometimes other ingredients
- Sopes: A round of fried corn masa with savory toppings
- Taco: fried and folded or rolled tortilla filled usually with chopped meat, and often a hot, spicy sauce. It may contain other ingredients such as shredded vegetables, cheese, etc.
- Tamal: mexican dish consisting of cornmeal dough around a filling as of minced meat and often a hot, spicy sauce. It may contain other ingredients such as, cheese, red peppers, etc. It is then wrapped in corn husks or plantain leaves and cooked by baking, steaming, etc.
- Torta: It is a kind of sandwich, but made with a large baked roll similar to a baguette but shorter in length.
- Tortilla: a thin, flat, round bread made of corn or wheat flour and baked on top of a stove
