# Supplementary material for: Design and Validation of a Food Frequency Questionnaire to Evaluate the Consumption of Trans Fatty Acids in the Adult Population (FFQ-TFA)
Source: Int J Environ Res Public Health. 2022 Oct 12;19(20):13097. doi: 10.3390/ijerph192013097 (PMC9602579; doi:10.3390/ijerph192013097)
Supplement: Supplementary file 1 [file ijerph-19-13097-s001.zip › Supplementary File S1_Español_2022_10_04.pdf]

Universidad Autónoma de Querétaro.
Facultad de Ciencias Naturales.
Maestría en Nutrición Clínica Integral.
Folio:______________________

Supplementary File S1

Cuestionarios de frecuencia alimentaria y prácticas alimentarias para estimar el consumo de ácidos grasos trans (FFQ-TFA y EPQ-TFA)

<table>
<tr><td colspan="6">Datos personales</td></tr>
<tr><td colspan="6">1.1 Nombre:____
Apellido paterno   Apellido materno   Nombre (s)</td></tr>
<tr><td>1.2 Sexo: (____)
Mujer = 1
Hombre =2</td><td>1.3 Edad (años):</td><td colspan="2">1.4 Teléfono:</td><td colspan="2">1.5 Fecha:_____/_____/____
Día / Mes / Año</td></tr>
<tr><td>1.6 Licenciatura: (____)
Administración (1)
Comercio Internacional (2)
Contador (3)</td><td>1.7 Semestre: (____)</td><td colspan="4">1.8 Estado Civil: (____)
Soltero (1)
Casado (2)
Divorciado (3)
Viudo (4)</td></tr>
<tr><td colspan="2">1.9 Vive con: (____)
Familia (1)
Amigos (2)
Pareja (3)
Sólo (4)</td><td colspan="4">1.12 IMC:</td></tr>
<tr><td>Peso:</td><td>1.10 Promedio</td><td>Talla (estatura):</td><td>1.11 Promedio</td><td>1.13 % de grasa corporal</td><td>Promedio</td></tr>
<tr><td>1:</td><td rowspan="2"></td><td>1:</td><td rowspan="2"></td><td>1:</td><td rowspan="2"></td></tr>
<tr><td>2:</td><td>2:</td><td>2:</td></tr>
</table>

I. Food frequency questionnaire (FFQ-TFA). Cuestionario de frecuencia de alimentos (FFQ-TFA)

Universidad Autónoma de Querétaro.
Facultad de Ciencias Naturales.
Maestría en Nutrición Clínica Integral.
Folio:________________________

PARTE 1.

Instrucciones de llenado:

1.- Inicia llenando el apartado de la frecuencia con los alimentos de la lista que consumiste en el último mes.

2.- No dejes en blanco ningún alimento, si no lo consumes elige la casilla "nunca".

3.- Responde lo más preciso posible, tanto los alimentos que consumes solos como aquellos que añades a los platillos.

4.- Elige sólo una respuesta por grupo de columnas. Marca con una X la casilla correspondiente al número de días en el mes o en la semana en que consumes el alimento, luego marca con otra X la casilla correspondiente a las veces que consumes el alimento por cada día.

5.- En la columna "número de porciones consumidas", marca con una X el número 1, 2, 3, 4 o 5 para indicar el número de porciones que consumes cada vez.

6.- Para ayudarte a rellenar las tablas, se te mostrará una serie de imágenes de los alimentos incluidos.

En promedio ¿Con qué frecuencia consumes los siguientes alimentos?

1.- Cereales

		Veces por mes o semana							Veces al día				
Alimento	Porción	Nunca (0)	1 vez por mes (1)	2-3 veces al mes (2)	1 vez por sema na (3)	2-4 veces por sema na (4)	5-6 veces por sema na (5)	Todos los días (6)	1 vez al día. (7)	2-3 veces al día (8)	4-5 veces al día (9)	+6 veces al día (10)	Número de porciones consumidas
1.1Bolillo	1 pieza mediana (70 g)	0	1	2	3	4	5	6	7	8	9	10	1 I 2 I 3 I 4 I5
1.2Pan de caja (tipo: Bimbo, Wonder, etc.)	1 rebanada (26 g)	0	1	2	3	4	5	6	7	8	9	10	1 I 2 I 3 I 4 I5
1.3Pan de caja tostado (tipo: Bimbo, Wonder etc.)	1 rebanada (21 g)	0	1	2	3	4	5	6	7	8	9	10	1 I 2 I 3 I 4 I5
1.4Galleta salada (tipo: saladitas)	5 cuadritos (16 g)	0	1	2	3	4	5	6	7	8	9	10	1 I 2 I 3 I 4 I5
1.5Baguette	1/3 de pieza (70 g)	0	1	2	3	4	5	6	7	8	9	10	1 I 2 I 3 I 4 I5
1.6Bollo	1 pieza (60 g)	0	1	2	3	4	5	6	7	8	9	10	1 I 2 I 3 I 4 I5
1.7Medias noches	1 pieza (25 g)	0	1	2	3	4	5	6	7	8	9	10	1 I 2 I 3 I 4 I5
1.8Tortilla de harina de trigo	3 piezas (75 g)	0	1	2	3	4	5	6	7	8	9	10	1 I 2 I 3 I 4 I5
1.9Tortilla de maíz	3 piezas promedio (100 g)	0	1	2	3	4	5	6	7	8	9	10	1 I 2 I 3 I 4 I5
1.10Arroz cocido	1 taza (50 g)	0	1	2	3	4	5	6	7	8	9	10	1 I 2 I 3 I 4 I5
1.11Cereal para desayuno (sin leche)	½ taza (44 g)	0	1	2	3	4	5	6	7	8	9	10	1 I 2 I 3 I 4 I5

Alimento	Porción	Nunca (0)	1 vez por mes (1)	2-3 veces al mes (2)	1 vez por semana (3)	2-4 veces por semana (4)	5-6 veces por semana (5)	Todos los días (6)	1 vez al día. (7)	2-3 veces al día (8)	4-5 veces al día (9)	+6 veces al día (10)	Número de porciones consumidas
**2.-Leche y derivados**													
2.1Leche entera líquida	1 taza (216 g)	0	1	2	3	4	5	6	7	8	9	10	1 I 2 I 3 I 4 I5
2.2Leche semidescremada líquida	1 taza (247 g)	0	1	2	3	4	5	6	7	8	9	10	1 I 2 I 3 I 4 I5
2.3Leche en polvo	2 cucharadas soperas copeteadas (18 g)	0	1	2	3	4	5	6	7	8	9	10	1 I 2 I 3 I 4 I5
2.4Leche evaporada preparada (tipo: Clavel, Sello Rojo, Nestlé)	1 taza (240 g)	0	1	2	3	4	5	6	7	8	9	10	1 I 2 I 3 I 4 I5
2.5Yogur natural o con fruta	¾ de taza o un vaso de presentación comercial (156 g)	0	1	2	3	4	5	6	7	8	9	10	1 I 2 I 3 I 4 I5
2.6Crema agria (tipo: Alpura, Lala, Great Value, etc.)	1 cucharada sopera copeteada (15 g)	0	1	2	3	4	5	6	7	8	9	10	1 I 2 I 3 I 4 I5
2.7Queso panela	1 rebanada mediana (30 g)	0	1	2	3	4	5	6	7	8	9	10	1 I 2 I 3 I 4 I5
2.8Queso Oaxaca	1 rebanada mediana (30 g)	0	1	2	3	4	5	6	7	8	9	10	1 I 2 I 3 I 4 I5
2.9Queso manchego	1 rebanada mediana (30 g)	0	1	2	3	4	5	6	7	8	9	10	1 I 2 I 3 I 4 I5
2.10Queso crema	1 cucharada copeteada (15 g)	0	1	2	3	4	5	6	7	8	9	10	1 I 2 I 3 I 4 I5
2.11 Queso doble crema	1 rebanada mediana (30 g)	0	1	2	3	4	5	6	7	8	9	10	1 I 2 I 3 I 4 I5
**3.- Productos de origen animal**													
3.1Carne de res	1 pieza de bistec promedio (90 g)	0	1	2	3	4	5	6	7	8	9	10	1 I 2 I 3 I 4 I5
3.2Carne de cerdo	1 pieza de bistec pequeño (60 g)	0	1	2	3	4	5	6	7	8	9	10	1 I 2 I 3 I 4 I5
3.3Pollo (pierna, muslo, pechuga)	1 pieza mediana (90 g)	0	1	2	3	4	5	6	7	8	9	10	1 I 2 I 3 I 4 I5
3.4Huevo (completo)	1 pieza (62 g)	0	1	2	3	4	5	6	7	8	9	10	1 I 2 I 3 I 4 I5
3.5Pescado fresco	1 filete mediano (90 g)	0	1	2	3	4	5	6	7	8	9	10	1 I 2 I 3 I 4 I5
3.6Atún enlatado drenado	½ lata (46 g)	0	1	2	3	4	5	6	7	8	9	10	1 I 2 I 3 I 4 I5
3.7 Sardina	1/3 de lata (70 g)	0	1	2	3	4	5	6	7	8	9	10	1 I 2 I 3 I 4 I5
3.8Mariscos (camarón, pulpo, etc)	½ taza (60 g)	0	1	2	3	4	5	6	7	8	9	10	1 I 2 I 3 I 4 I5

Alimento	Porción	Nunca (0)	1 vez por mes (1)	2-3 veces al mes (2)	1 vez por semana (3)	2-4 veces por semana (4)	5-6 veces por semana (5)	Todos los días (6)	1 vez al día. (7)	2-3 veces al día (8)	4-5 veces al día (9)	+6 veces al día (10)	Número de porciones consumidas
3.9Salchicha	2 piezas (46 g)	0	1	2	3	4	5	6	7	8	9	10	1 I 2 I 3 I 4 I5
3.10Jamón	1 rebanada mediana (16 g)	0	1	2	3	4	5	6	7	8	9	10	1 I 2 I 3 I 4 I5
3.11Chorizo	1 trozo mediano (41 g)	0	1	2	3	4	5	6	7	8	9	10	1 I 2 I 3 I 4 I5
3.12Mortadela	1 rebanada mediana (30 g)	0	1	2	3	4	5	6	7	8	9	10	1 I 2 I 3 I 4 I5
**4.- Repostería y panadería**													
4.1Cuerno de hojaldre	1 pieza mediana (70 g)	0	1	2	3	4	5	6	7	8	9	10	1 I 2 I 3 I 4 I5
4.2Concha de panadería	1 pieza mediana (70 g)	0	1	2	3	4	5	6	7	8	9	10	1 I 2 I 3 I 4 I5
4.3Concha industrializada	1 pieza (70 g)	0	1	2	3	4	5	6	7	8	9	10	1 I 2 I 3 I 4 I5
4.4Dona de panadería	1 pieza (70 g)	0	1	2	3	4	5	6	7	8	9	10	1 I 2 I 3 I 4 I5
4.5Dona glaseada industrializada	3 piezas (52 g)	0	1	2	3	4	5	6	7	8	9	10	1 I 2 I 3 I 4 I5
4.6Dona azucarada industrializada	2 piezas (52 g)	0	1	2	3	4	5	6	7	8	9	10	1 I 2 I 3 I 4 I5
4.7Panqué de panadería	1 rebanada grande (64 g)	0	1	2	3	4	5	6	7	8	9	10	1 I 2 I 3 I 4 I5
4.8Panqué industrializado	1 rebanada mediana (32 g)	0	1	2	3	4	5	6	7	8	9	10	1 I 2 I 3 I 4 I5
4.9Buñuelos	2 piezas (33 g)	0	1	2	3	4	5	6	7	8	9	10	1 I 2 I 3 I 4 I5
4.10Pan banderilla	1 pieza mediana (80 g)	0	1	2	3	4	5	6	7	8	9	10	1 I 2 I 3 I 4 I5
4.11Galleta dulces sin relleno (tipo: avena, amaranto, etc.)	2 piezas (40 g)	0	1	2	3	4	5	6	7	8	9	10	1 I 2 I 3 I 4 I5
4.12Galleta con relleno (tipo: Emperador, Plativolos, Oreo, Príncipe, etc.)	5 piezas (57 g)	0	1	2	3	4	5	6	7	8	9	10	1 I 2 I 3 I 4 I5
4.13Galleta de chocolate o mantequilla (tipo: Pastisetas)	4 piezas (30 g)	0	1	2	3	4	5	6	7	8	9	10	1 I 2 I 3 I 4 I5
4.14Galleta con chispas de chocolate (tipo: Chokis, Triki-trakes, Chips ahoy, etc.)	6 piezas (57 g)	0	1	2	3	4	5	6	7	8	9	10	1 I 2 I 3 I 4 I5
4.15Galleta de nieve (tipo: Suavicremas, Cremax, etc.)	4 piezas (44 g)	0	1	2	3	4	5	6	7	8	9	10	1 I 2 I 3 I 4 I5
4.16Galleta salada grasosa (tipo: Crakets, Ritz, etc.)	12 piezas (35 g)	0	1	2	3	4	5	6	7	8	9	10	1 I 2 I 3 I 4 I5

Alimento	Porción	Nunca (0)	1 vez por mes (1)	2-3 veces al mes (2)	1 vez por semana (3)	2-4 veces por semana (4)	5-6 veces por semana (5)	Todos los días (6)	1 vez al día. (7)	2-3 veces al día (8)	4-5 veces al día (9)	+6 veces al día (10)	Número de porciones consumidas
4.17Galleta dulces (tipo: marías, animalitos, etc.)	12 piezas (50 g)	0	1	2	3	4	5	6	7	8	9	10	1 I 2 I 3 I 4 I5
4.18Polvorón	4 piezas (60 g)	0	1	2	3	4	5	6	7	8	9	10	1 I 2 I 3 I 4 I5
4.1Oreja	1 pieza mediana (70 g)	0	1	2	3	4	5	6	7	8	9	10	1 I 2 I 3 I 4 I5
4.20Barrita	2 piezas medianas (55 g)	0	1	2	3	4	5	6	7	8	9	10	1 I 2 I 3 I 4 I5
4.21Rol de canela de panadería	1 pieza mediana (70 g)	0	1	2	3	4	5	6	7	8	9	10	1 I 2 I 3 I 4 I5
4.22Rol de canela industrializado	1 pieza mediana (70 g)	0	1	2	3	4	5	6	7	8	9	10	1 I 2 I 3 I 4 I5
4.23Mantecada (tipo: Bimbo, Tía Rosa, etc.)	2 piezas medianas (60 g)	0	1	2	3	4	5	6	7	8	9	10	1 I 2 I 3 I 4 I5
4.24Tiramisú	½ pieza (41 g)	0	1	2	3	4	5	6	7	8	9	10	1 I 2 I 3 I 4 I5
4.25Pastel de chocolate o zanahoria	1 rebanada grande (163 g)	0	1	2	3	4	5	6	7	8	9	10	1 I 2 I 3 I 4 I5
4.26Pastelillo industrializado (tipo: Gansito, Pingüino, Negrito, Submarino, Chocorol, etc.)	1 pieza (59 g)	0	1	2	3	4	5	6	7	8	9	10	1 I 2 I 3 I 4 I5
4.27Barra de cereal (tipo: Special K, All bran , Bran frut, etc.)	1 pieza pequeña (25 g)	0	1	2	3	4	5	6	7	8	9	10	1 I 2 I 3 I 4 I5
**5.- Botanas**													
5.1Fritura de maíz industrializada (tipo: Fritos, Takis, Churrumais, Runners, etc.)	1 paquete mediano (46 g)	0	1	2	3	4	5	6	7	8	9	10	1 I 2 I 3 I 4 I5
5.2Papas fritas industrializadas (tipo: Sabritas, Chips, Pringles, Ruffles, etc.)	1 paquete mediano (45 g)	0	1	2	3	4	5	6	7	8	9	10	1 I 2 I 3 I 4 I5
5.3Palomitas para microondas	½ paquete (30 g)	0	1	2	3	4	5	6	7	8	9	10	1 I 2 I 3 I 4 I5

Universidad Autónoma de Querétaro.
Facultad de Ciencias Naturales.
Maestría en Nutrición Clínica Integral.
Folio:____________________________

Alimento	Porción	Nunca (0)	1 vez por mes (1)	2-3 veces al mes (2)	1 vez por semana (3)	2-4 veces por semana (4)	5-6 veces por semana (5)	Todos los días (6)	1 vez al día. (7)	2-3 veces al día (8)	4-5 veces al día (9)	+6 veces al día (10)	Número de porciones consumidas
5.4Plátanos fritos industrializados	1 paquete mediano (32 g)	0	1	2	3	4	5	6	7	8	9	10	1 I 2 I 3 I 4 I5
**6.- Comida preparada**													
6.1Antojitos mexicanos: sopes, quesadillas, gorditas, tacos dorados.	2 piezas (144 g)	0	1	2	3	4	5	6	7	8	9	10	1 I 2 I 3 I 4 I5
6.2Enchiladas servidas	3 piezas (150 g)	0	1	2	3	4	5	6	7	8	9	10	1 I 2 I 3 I 4 I5
6.3Taco de barbacoa	1 pieza (70 g)	0	1	2	3	4	5	6	7	8	9	10	1 I 2 I 3 I 4 I5
6.4Tamal	1 pieza grande (200 g)	0	1	2	3	4	5	6	7	8	9	10	1 I 2 I 3 I 4 I5
6.5Papas fritas caseras	1 taza (75 g)	0	1	2	3	4	5	6	7	8	9	10	1 I 2 I 3 I 4 I5
6.6Chicharrón de harina frito	1 bolsa (18 g)	0	1	2	3	4	5	6	7	8	9	10	1 I 2 I 3 I 4 I5
6.7Torta completa	1 pieza mediana (150 g)	0	1	2	3	4	5	6	7	8	9	10	1 I 2 I 3 I 4 I5
6.8 Empanada	1 pieza grande (120 g)	0	1	2	3	4	5	6	7	8	9	10	1 I 2 I 3 I 4 I5
6.9Hot cake	2 piezas medianas (120 g)	0	1	2	3	4	5	6	7	8	9	10	1 I 2 I 3 I 4 I5
6.10Churro con azúcar	1 pieza (100 g)	0	1	2	3	4	5	6	7	8	9	10	1 I 2 I 3 I 4 I5
**7.- Comida rápida**													
7.1Papas a la francesa industrializadas	1 paquete (53 g)	0	1	2	3	4	5	6	7	8	9	10	1 I 2 I 3 I 4 I5
7.2Pollo frito (tipo: KFC)	2 piezas medianas (108 g)	0	1	2	3	4	5	6	7	8	9	10	1 I 2 I 3 I 4 I5
7.3 Hamburguesa de res	1 pieza mediana (160 g)	0	1	2	3	4	5	6	7	8	9	10	1 I 2 I 3 I 4 I5
7.4Hamburguesa de pollo	1 pieza mediana (160 g)	0	1	2	3	4	5	6	7	8	9	10	1 I 2 I 3 I 4 I5
7.5Pizza	1 rebanada mediana (135 g)	0	1	2	3	4	5	6	7	8	9	10	1 I 2 I 3 I 4 I5
7.6Burrito	1 pieza mediana (120 g)	0	1	2	3	4	5	6	7	8	9	10	1 I 2 I 3 I 4 I5
7.7Banderilla	1 pieza (120 g)	0	1	2	3	4	5	6	7	8	9	10	1 I 2 I 3 I 4 I5
**8.- Aceites y grasas**													
8.1Mayonesa	1 cucharada sopera copeteada (9 g)	0	1	2	3	4	5	6	7	8	9	10	1 I 2 I 3 I 4 I5
8.2Mantequilla	1 cucharada cafetera (5 g)	0	1	2	3	4	5	6	7	8	9	10	1 I 2 I 3 I 4 I5
8.3Margarina	1 cucharada cafetera (5 g)	0	1	2	3	4	5	6	7	8	9	10	1 I 2 I 3 I 4 I5

Alimento	Porción	Nunca (0)	1 vez por mes (1)	2-3 veces al mes (2)	1 vez por semana (3)	2-4 veces por semana (4)	5-6 veces por semana (5)	Todos los días (6)	1 vez al día. (7)	2-3 veces al día (8)	4-5 veces al día (9)	+6 veces al día (10)	Número de porciones consumidas
8.4Crema de cacahuate	1 cucharada sopera copeteada (27 g)	0	1	2	3	4	5	6	7	8	9	10	1 I 2 I 3 I 4 I5
8.5Aceite de oliva	1 cucharada sopera (10 g)	0	1	2	3	4	5	6	7	8	9	10	1 I 2 I 3 I 4 I5
8.6Cacahuates y semillas de calabaza	½ paquete mediano (35 g)	0	1	2	3	4	5	6	7	8	9	10	1 I 2 I 3 I 4 I5
8.7Aguacate	1/3 de pieza (37 g)	0	1	2	3	4	5	6	7	8	9	10	1 I 2 I 3 I 4 I5
9.-Alimentos dulces													
9.1Chocolate en barra (tipo: Carlos V, Larín , Vaquita, Hershey´s, etc.)	1 barra pequeña (23 g)	0	1	2	3	4	5	6	7	8	9	10	1 I 2 I 3 I 4 I5
9.2Chocolate con galleta (tipo: Tin-larín, Bocadín, Kit-kat, etc.)	1 pieza mediana (33 g)	0	1	2	3	4	5	6	7	8	9	10	1 I 2 I 3 I 4 I5
9.3Chocolate confitado (tipo: M&M, lunetas, etc.)	1 paquete pequeño (24 g)	0	1	2	3	4	5	6	7	8	9	10	1 I 2 I 3 I 4 I5
9.4Dulce con consistencia chiclosa	2 piezas pequeñas (7 g)	0	1	2	3	4	5	6	7	8	9	10	1 I 2 I 3 I 4 I5
9.5Leche condensada	1 cucharada sopera (19 g)	0	1	2	3	4	5	6	7	8	9	10	1 I 2 I 3 I 4 I5

Universidad Autónoma de Querétaro.
Facultad de Ciencias Naturales.
Maestría en Nutrición Clínica Integral.
Folio:________________________

PARTE 2. CONSUMO DE GRASAS

	**¿Qué tipo de grasa usas para preparar los siguientes guisados?**<br><br>PARA CADA PREPARACIÓN, MARCA 1 SI LA GRASA ES USADA Y 0 SI NO ES USADA								**Usualmente, como prepara estos alimentos.**<br>**Asado (1)**<br>**Cocinado (2)**<br>**Frito (3)**
**Preparaciones**	Aceite vegetal (1)	Manteca de cerdo (2)	Manteca vegetal (3)	Margarina (4)	Mantequilla (5)	Aceite en aerosol (6)	No usa grasa (7)	No consume la preparación (8)	
2.1 Arroz guisado	|___|	|___|	|___|	|___|	|___|	|___|	|___|	|___|	|___|
2.2 Sopa de pasta frita	|___|	|___|	|___|	|___|	|___|	|___|	|___|	|___|	|___|
2.3 Frijoles de olla	|___|	|___|	|___|	|___|	|___|	|___|	|___|	|___|	|___|
2.4 Frijoles refritos	|___|	|___|	|___|	|___|	|___|	|___|	|___|	|___|	|___|
2.5 Plátanos fritos	|___|	|___|	|___|	|___|	|___|	|___|	|___|	|___|	|___|
2.6 Huevos estrellados	|___|	|___|	|___|	|___|	|___|	|___|	|___|	|___|	|___|
2.7 Huevos revueltos	|___|	|___|	|___|	|___|	|___|	|___|	|___|	|___|	|___|
2.8 Pollo	|___|	|___|	|___|	|___|	|___|	|___|	|___|	|___|	|___|
2.9 Res	|___|	|___|	|___|	|___|	|___|	|___|	|___|	|___|	|___|
2.10 Pescado	|___|	|___|	|___|	|___|	|___|	|___|	|___|	|___|	|___|
2.11 Verduras capeadas	|___|	|___|	|___|	|___|	|___|	|___|	|___|	|___|	|___|
2.12 Tortitas de papa y papas fritas	|___|	|___|	|___|	|___|	|___|	|___|	|___|	|___|	|___|
2.13 Tortitas de carne o pollo capeadas	|___|	|___|	|___|	|___|	|___|	|___|	|___|	|___|	|___|
2.14 Empanizados (pollo o carnes rojas)	|___|	|___|	|___|	|___|	|___|	|___|	|___|	|___|	|___|
3.15 Antojitos mexicanos (quesadillas, tacos, flautas, gorditas, sopes, tamales)	|___|	|___|	|___|	|___|	|___|	|___|	|___|	|___|	|___|

*NOTA: SI USAS MÁS DE UN TIPO DE GRASA EN CADA GUISADO, MARCA LAS CASILLAS CORRESPONDIENTES

Universidad Autónoma de Querétaro.
Facultad de Ciencias Naturales.
Maestría en Nutrición Clínica Integral.
Folio:________________________

CONSUMO DE SAZONADOR

	**¿Cada cuando usas Sazonador tipo knorr para preparar los siguientes guisados?**<br><br>PARA CADA PREPARACIÓN, MARCA 1 en la frecuencia con que lo usas. Si no consumes esa preparación llena con 1 el no aplica						
**Preparaciones**	No Aplica	No usa	Usa poco (menos de 1 vez por semana)	Usa algunas veces por semana (2-3 veces)	Usa frecuentemente (4-6 veces)	Usa todos los días	No sabe
3.1 Arroz guisado	|___|	|___|	|___|	|___|	|___|	|___|	|___|
3.2 Sopa de pasta frita	|___|	|___|	|___|	|___|	|___|	|___|	|___|
3.3 Frijoles de olla	|___|	|___|	|___|	|___|	|___|	|___|	|___|
3.4 Frijoles refritos	|___|	|___|	|___|	|___|	|___|	|___|	|___|
3.5 Plátanos fritos	|___|	|___|	|___|	|___|	|___|	|___|	|___|
3.6 Huevos estrellados	|___|	|___|	|___|	|___|	|___|	|___|	|___|
3.7 Huevos revueltos	|___|	|___|	|___|	|___|	|___|	|___|	|___|
3.8 Pollo	|___|	|___|	|___|	|___|	|___|	|___|	|___|
3.9 Res	|___|	|___|	|___|	|___|	|___|	|___|	|___|
3.10 Pescado	|___|	|___|	|___|	|___|	|___|	|___|	|___|
3.11 Verduras capeadas	|___|	|___|	|___|	|___|	|___|	|___|	|___|
3.12 Tortitas de papa y papas fritas	|___|	|___|	|___|	|___|	|___|	|___|	|___|
3.13 Tortitas de carne o pollo capeadas	|___|	|___|	|___|	|___|	|___|	|___|	|___|
3.14 Empanizados (pollo o carnes rojas)	|___|	|___|	|___|	|___|	|___|	|___|	|___|
4.15 antojitos mexicanos (quesadillas, tacos, flautas, gorditas, sopes, tamales)	|___|	|___|	|___|	|___|	|___|	|___|	|___|

II. Cuestionario de prácticas alimentarias relacionadas con el consumo de ácidos grasos trans (EPQ-TFA)

Instrucciones de llenado: elige una opción de respuesta para cada una de las preguntas, a menos que la pregunta indique que se pueden elegir varias opciones. Circular la respuesta que responda mejor a la pregunta de acuerdo a las actividades que realiza.

1. ¿Con que frecuencia comes fuera de casa?
   a. Todos los días (5)
   b. 5-6 veces a la semana (4)
   c. 3-4 veces a la semana (3)
   d. 1-2 veces a la semana (2)
   e. 1-2 veces al mes (1)
   f. Nunca (0)

2. ¿Bajo qué método son preparados los alimentos que consumes fuera de casa en la mayoría de las ocasiones?
   a. Fritura (4)
   b. Guiso (3)
   c. Asado (2)
   d. Horneado o a la plancha (1)
   e. Hervido (0)
   f. Al vapor (0)

3. ¿Qué tipo de grasa utilizas para cocinar en la mayoría de las ocasiones?
   a. Margarina (5)
   b. Manteca vegetal (4)
   c. Manteca de cerdo (3)
   d. Mantequilla (2)
   e. Aceite vegetal (1)
   f.

4. ¿Reutilizas el aceite para cocinar?
   a. Siempre (2)
   b. A veces (1)
   c. Nunca (0)

5. ¿Consumes alimentos evitando frituras y grasas tanto en casa como fuera de la misma?
    a. Siempre (2)
    b. A veces (1)
    c. Nunca (0)

6. ¿Con qué frecuencia revisas las etiquetas de los alimentos que consumes para verificar el contenido de grasa?
    a. Nunca (3)
    b. A veces (2)
    c. Frecuentemente (1)
    d. Siempre (0)

7. Cuando revisas las etiquetas ¿logras comprenderlas completamente?
    a. Nunca (3)
    b. A veces (2)
    c. Frecuentemente (1)
    d. Siempre (0)

8. ¿Qué haces con la grasa que puede verse a simple vista en los alimentos tanto líquidos como sólidos?
    a. No quito nada (2)
    b. Quito un poco (1)
    c. La quito toda (0)

9. ¿Qué consumes entre comidas? (en la mayoría de las ocasiones)
    a. Galletas o pan dulce (3)
    b. Papas fritas, churros o frituras (2)
    c. Cacahuates o semillas (1)
    d. Dulces (0)
    e. Fruta o verdura (0)
    f. Ninguna de las anteriores(0)

10. ¿Con qué frecuencia consumes comida rápida? (pizza, hamburguesa, pollo frito, quesadillas fritas, sopes, gorditas etc.)
    a. Todos los días (6)
    b. 5-6 veces a la semana (5)

c. 3-4 veces a la semana(4)
d. 1-2 veces a la semana(3)
e. 2-3 veces al mes (2)
f. 1 vez al mes (1)
g. Nunca (0)

11. ¿Con qué frecuencia consumes galletas dulces?
   a. Todos los días (6)
   b. 5-6 veces a la semana (5)
   c. 3-4 veces a la semana(4)
   d. 1-2 veces a la semana(3)
   e. 2-3 veces al mes (2)
   f. 1 vez al mes (1)
   g. Nunca (0)

12. ¿Con qué frecuencia consumes pan dulce?
   a. Todos los días (6)
   b. 5-6 veces a la semana (5)
   c. 3-4 veces a la semana(4)
   d. 1-2 veces a la semana(3)
   e. 2-3 veces al mes (2)
   f. 1 vez al mes (1)
   g. Nunca (0)

13. ¿Con qué frecuencia consumes donas?
   a. Todos los días (6)
   b. 5-6 veces a la semana (5)
   c. 3-4 veces a la semana(4)
   d. 1-2 veces a la semana(3)
   e. 2-3 veces al mes (2)
   f. 1 vez al mes (1)
   g. Nunca (0)

14. ¿Con qué frecuencia consumes pasteles?
    a. Todos los días (6)
    b. 5-6 veces a la semana (5)
    c. 3-4 veces a la semana(4)
    d. 1-2 veces a la semana(3)
    e. 2-3 veces al mes (2)
    f. 1 vez al mes (1)
    g. Nunca (0)

15. ¿Con qué frecuencia consumes botanas fritas (chicharrones, frituras de maíz, frituras industrializadas)?
    a. Todos los días (6)
    b. 5-6 veces a la semana (5)
    c. 3-4 veces a la semana(4)
    d. 1-2 veces a la semana(3)
    e. 2-3 veces al mes (2)
    f. 1 vez al mes (1)
    g. Nunca (0)

Total de puntos:___________________________

Universidad Autónoma de Querétaro.
Facultad de Ciencias Naturales.
Maestría en Nutrición Clínica Integral.
Folio:__________________________

II. Eating practices questionnaire related to the consumption of trans fatty acids (EPQ-TFA)

Instructions: Sólo para uso del entrevistador

1. ¿Con que frecuencia comes fuera de casa?
    a. Todos los días (5)
    b. 5-6 veces a la semana (4)
    c. 3-4 veces a la semana (3)
    d. 1-2 veces a la semana (2)
    e. 1-2 veces al mes (1)
    f. Nunca (0)

a = 10
b = 8.3
c = 6.64
d = 4.98
e = 3.32
f = 1.66

2.-¿Bajo qué método son preparados los alimentos que consumes fuera de casa en la mayoría de las ocasiones?
a. Fritura (4)
b. Guiso (3)
c. Asado (2)
d. Horneado o a la plancha (1)
e. Hervido (0)
f. Al vapor (0)

a = 10
b = 8.3
c = 6.64
d = 4.98
e = 3.32
f = 1.66

3. ¿Qué tipo de grasa utilizas para cocinar en la mayoría de las ocasiones?
    a. Margarina (5)
    b. Manteca vegetal (4)
    c. Manteca de cerdo (3)
    d. Mantequilla (2)
    e. Aceite vegetal (1)

a = 10
b = 8
c = 6
d = 4
e = 2

4. ¿Reutilizas el aceite para cocinar?
    a. Siempre (2)
    b. A veces (1)
    c. Nunca (0)

a = 10
b = 6.66
c = 3.33

5. ¿Consumes alimentos evitando frituras y grasas tanto en casa como fuera de la misma?
   a. Siempre (2)
   b. A veces (1)
   c. Nunca (0)

a = 10
b = 6.66
c = 3.33

6. ¿Con qué frecuencia revisas las etiquetas de los alimentos que consumes para verificar el contenido de grasa?
   a. Nunca (3)
   b. A veces (2)
   c. Frecuentemente (1)
   d. Siempre (0)

a = 10
b = 7.5
c = 5
d = 2.5

7. Cuando revisas las etiquetas ¿logras comprenderlas completamente?
   a. Nunca (3)
   b. A veces (2)
   c. Frecuentemente (1)
   d. Siempre (0)

a = 10
b = 7.5
c = 5
d = 2.5

8. . ¿Qué haces con la grasa que puede verse a simple vista en los alimentos tanto líquidos como sólidos?
   a. No quito nada (2)
   b. Quito un poco (1)
   c. La quito toda (0)

a = 10
b = 6.66
c = 3.33

9. ¿Qué consumes entre comidas? (en la mayoría de las ocasiones)
   a. Galletas o pan dulce (3)
   b. Papas fritas, churros o frituras (2)
   c. Cacahuates o semillas (1)
   d. Dulces (0)
   e. Fruta o verdura (0)
   a. Ninguna de las anteriores(0)

a = 10
b = 8.52
c = 7.1
d = 5.68
e = 4.26
f = 2.84

10. ¿Con qué frecuencia consumes comida rápida? (pizza, hamburguesa, pollo frito, quesadillas fritas, sopes, gorditas etc.)
    a. Todos los días (6)
    b. 5-6 veces a la semana (5)
    c. 3-4 veces a la semana(4)
    d. 1-2 veces a la semana(3)
    e. 2-3 veces al mes (2)
    f. 1 vez al mes (1)

Nunca (0)

a = 10
b = 8.52
c = 7.1
d = 5.68
e = 4.26
f = 2.84
g =1.42

11.¿Con qué frecuencia consumes galletas dulces?
    a. Todos los días (6)
    b. 5-6 veces a la semana (5)
    c. 3-4 veces a la semana(4)
    d. 1-2 veces a la semana(3)
    e. 2-3 veces al mes (2)
    f. 1 vez al mes (1)
    g. Nunca (0)

a = 10
b = 8.52
c = 7.1
d = 5.68
e = 4.26
f = 2.84
g =1.42

12.¿Con qué frecuencia consumes pan dulce?
    a. Todos los días (6)
    b. 5-6 veces a la semana (5)
    c. 3-4 veces a la semana(4)
    d. 1-2 veces a la semana(3)
    e. 2-3 veces al mes (2)
    f. 1 vez al mes (1)
    g. Nunca (0)

a = 10
b = 8.52
c = 7.1
d = 5.68
e = 4.26
f = 2.84
g =1.42

Universidad Autónoma de Querétaro.
Facultad de Ciencias Naturales.
Maestría en Nutrición Clínica Integral.
Folio:______________________

13.¿Con qué frecuencia consumes donas?

a. Todos los días (6)
b. 5-6 veces a la semana (5)
c. 3-4 veces a la semana(4)
d. 1-2 veces a la semana(3)
e. 2-3 veces al mes (2)
f. 1 vez al mes (1)
g. Nunca (0)

a = 10
b = 8.52
c = 7.1
d = 5.68
e = 4.26
f = 2.84
g =1.42

14. ¿Con qué frecuencia consumes pasteles?

a. Todos los días (6)
b. 5-6 veces a la semana (5)
c. 3-4 veces a la semana(4)
d. 1-2 veces a la semana(3)
e. 2-3 veces al mes (2)
f. 1 vez al mes (1)
g. Nunca (0)

a = 10
b = 8.52
c = 7.1
d = 5.68
e = 4.26
f = 2.84
g =1.42

15.-¿Con qué frecuencia consumes botanas fritas (chicharrones, frituras de maíz, frituras industrializadas)?

a. Todos los días (6)
b. 5-6 veces a la semana (5)
c. 3-4 veces a la semana(4)
d. 1-2 veces a la semana(3)
e. 2-3 veces al mes (2)
f. 1 vez al mes (1)
g. Nunca (0)

a = 10
b = 8.52
c = 7.1
d = 5.68
e = 4.26
f = 2.84
g =1.42

Total de puntos:___________________________
