# Supplementary material for: Design and Validation of a Food Frequency Questionnaire to Evaluate the Consumption of Trans Fatty Acids in the Adult Population (FFQ-TFA)
Source: Int J Environ Res Public Health. 2022 Oct 12;19(20):13097. doi: 10.3390/ijerph192013097 (PMC9602579; doi:10.3390/ijerph192013097)
Supplement: Supplementary file 1 [file ijerph-19-13097-s001.zip › Supplementary File S2 Food Atlas.pdf]

Supplementary File S2

**Atlas de apoyo con imágenes de los alimentos contenidos en la Frecuencia de alimentos para estimar consumo de ácidos grasos trans.**<br>**Food Atlas as visual aid with foods contained in the Food frequency questionnaire to estimate the consumption of trans fatty acids**		
**1.1** 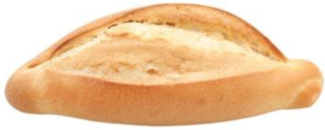	**1.2** 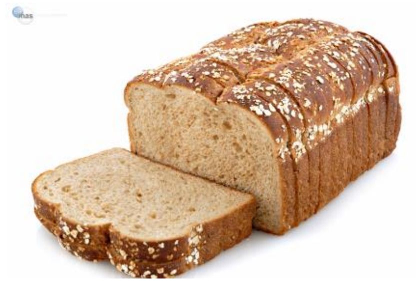	**1.3** 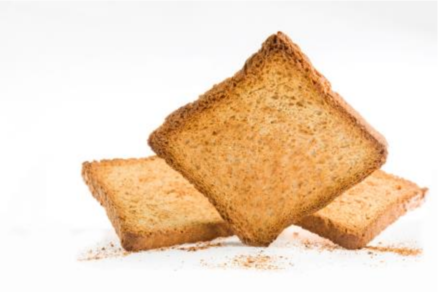
**1.4** 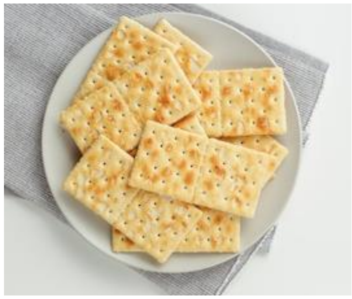	**1.5** 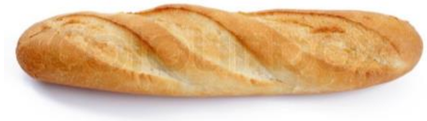	**1.6** 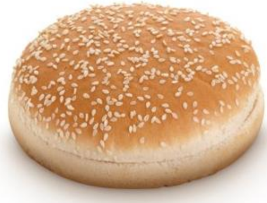
**1.7** 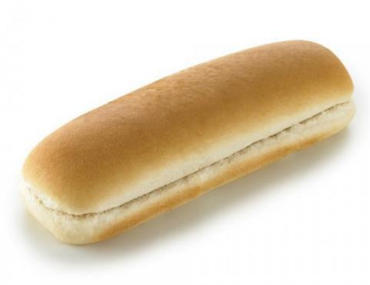	**1.8** 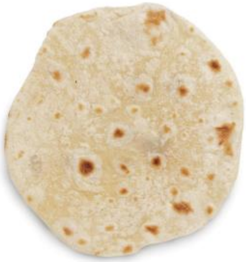	**1.9** 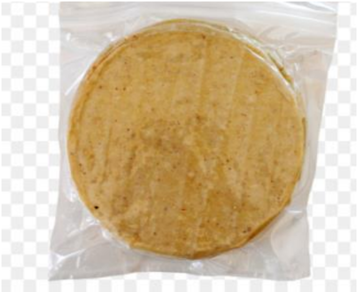

**1.10** 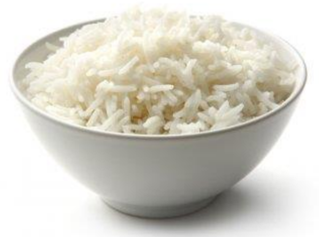	**1.11** 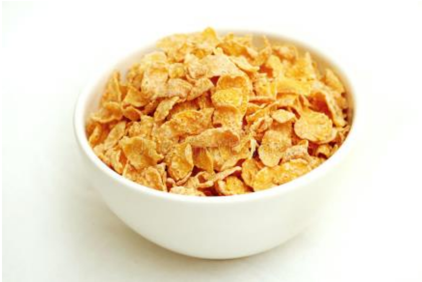	**2.1 y 2.2** 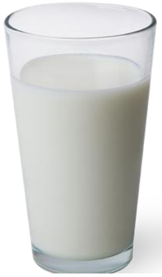
**2.3** 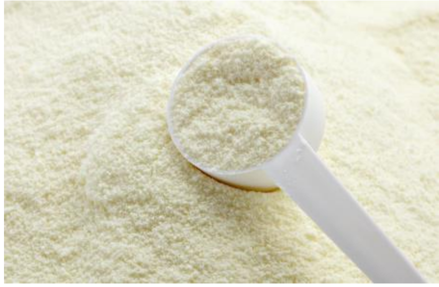	**2.4** 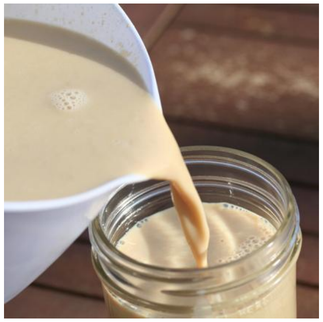	**2.5** 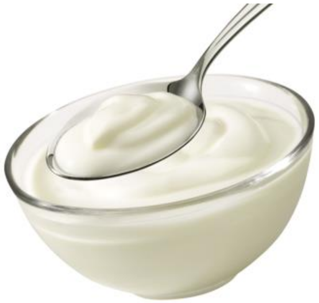
**2.6** 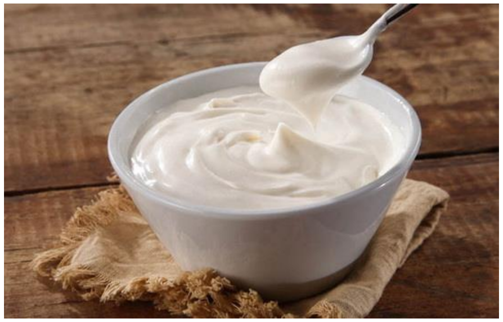	**2.7** 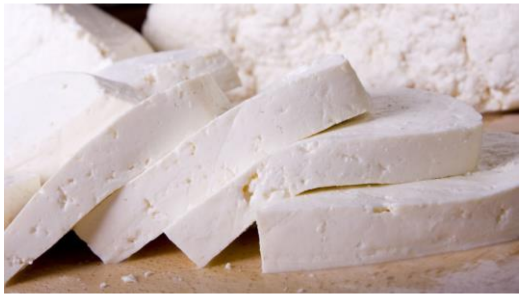	**2.8** 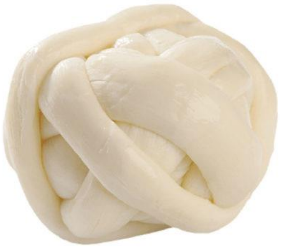

**2.9** 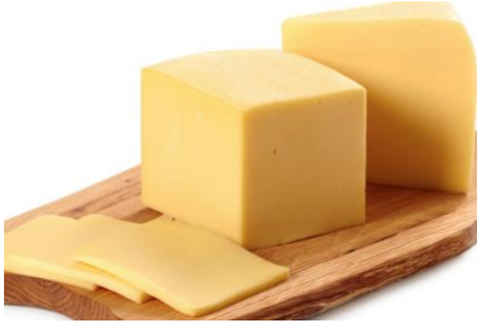	**2.10 y 2.11** 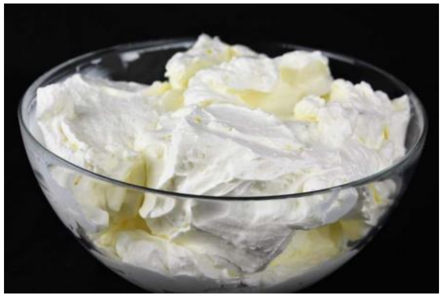	**3.1** 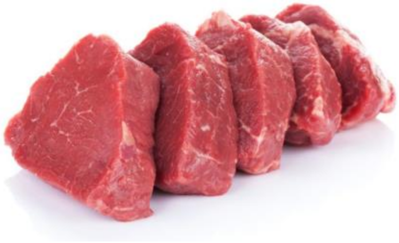
**3.2** 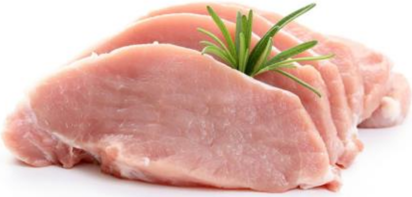	**3.3** 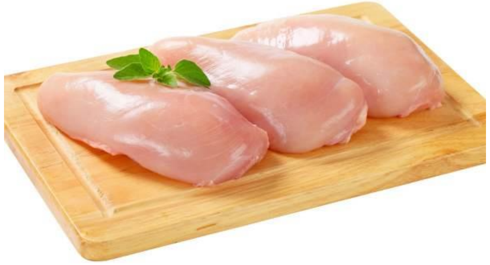	**3.4** 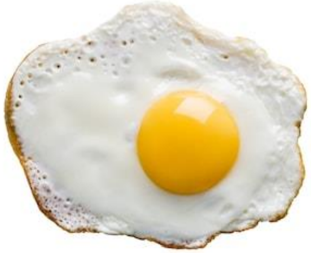
3.5 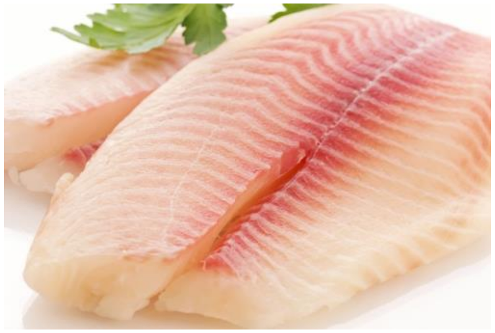	**3.6** 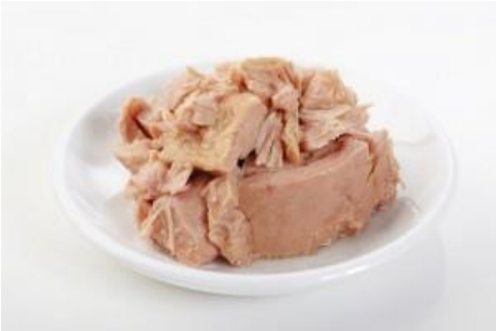	**3.7** 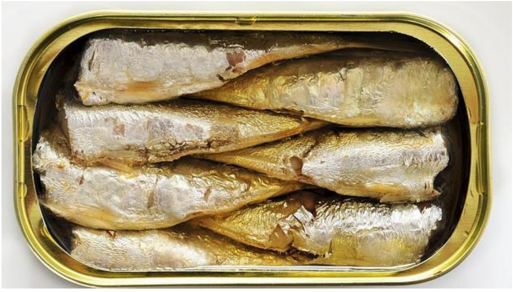

**3.8** 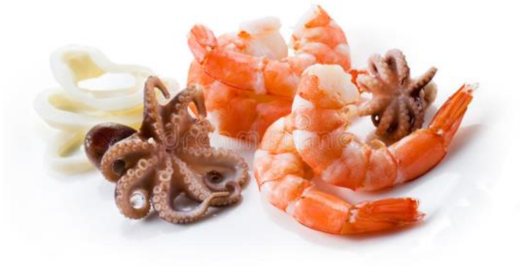	**3.9** 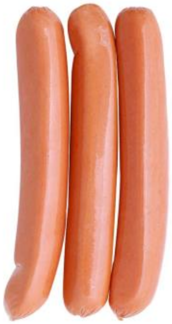	**3.10** 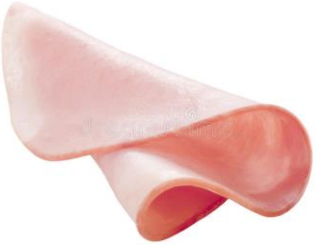
**3.11** 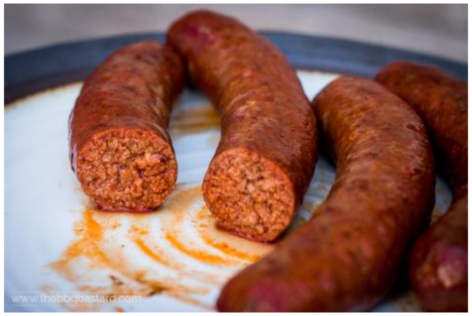	**3.12** 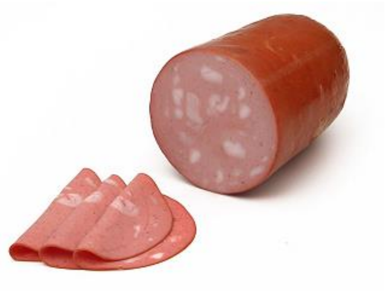	**4.1** 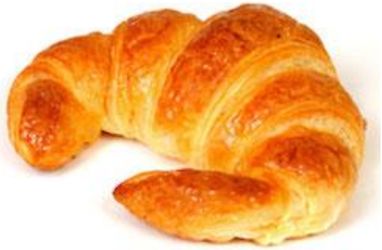
**4.2 y 4.3** 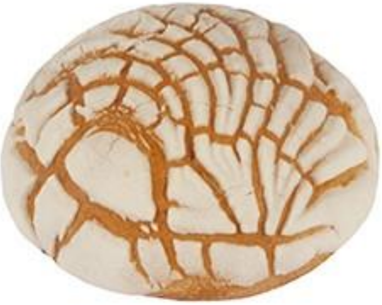	**4.4, 4.5 y 4.6** 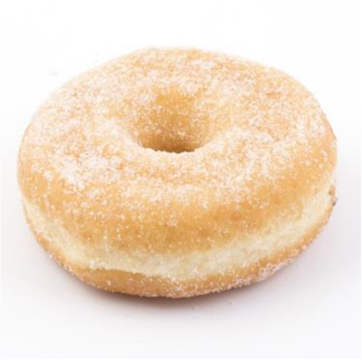	**4.7 y 4.8** 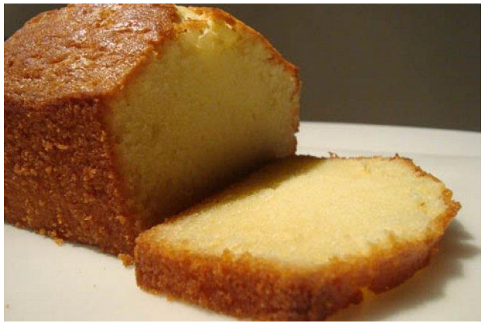

**4.9** 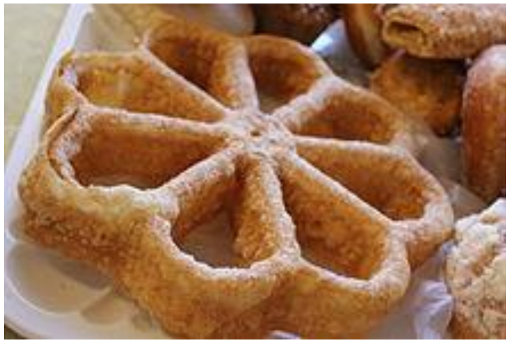	**4.10** 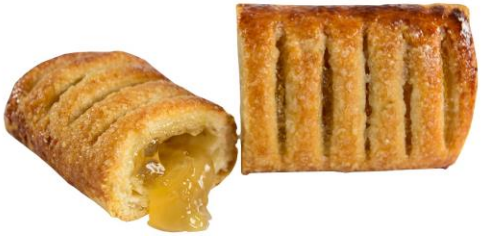	**4.11** 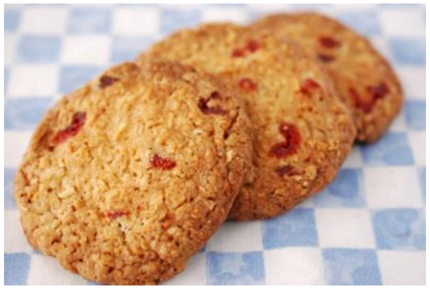
**4.12** 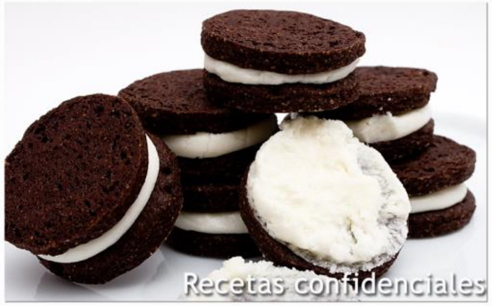	**4.13** 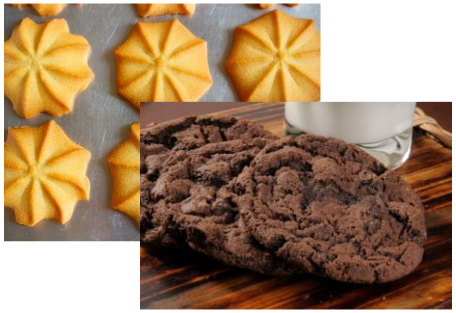	**4.14** 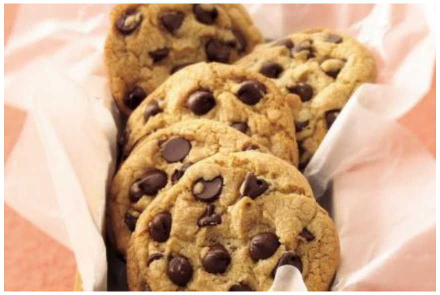
**4.15** 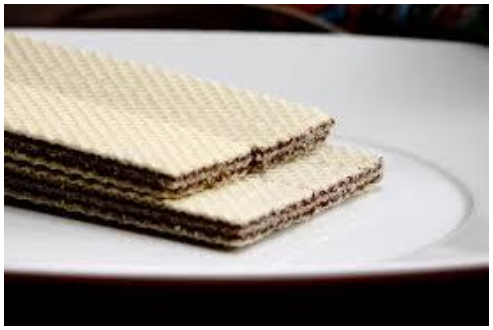	**4.16** 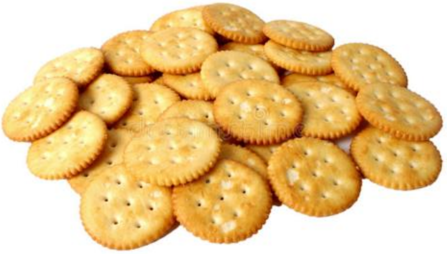	**4.17** 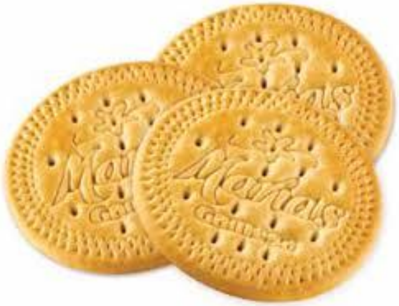

**4.18** 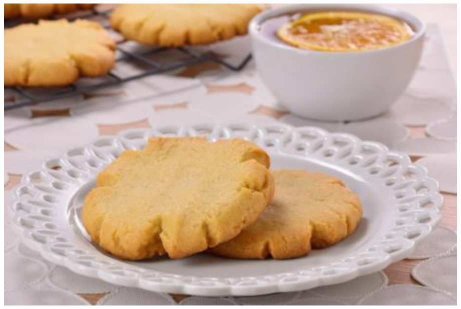	**4.19** 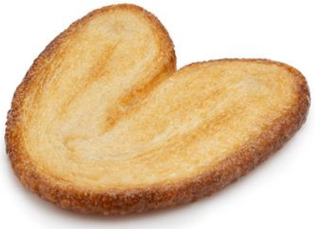	**4.20** 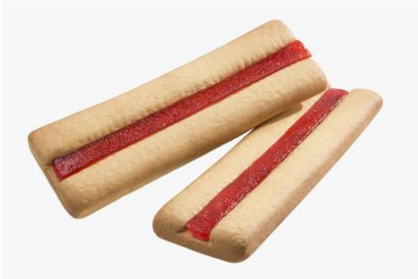
**4.21 y 4.22** 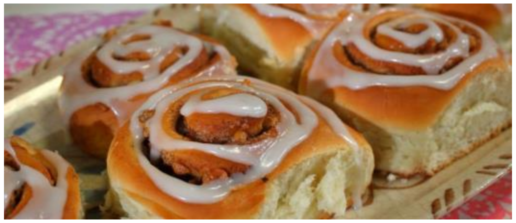	**4.23** 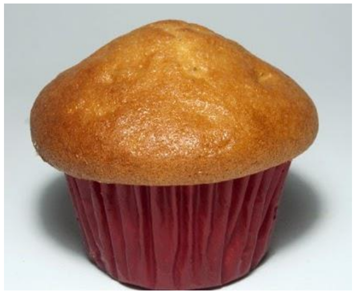	**4.24** 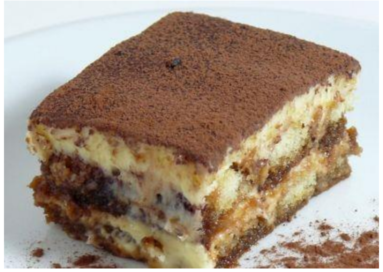
**4.25** 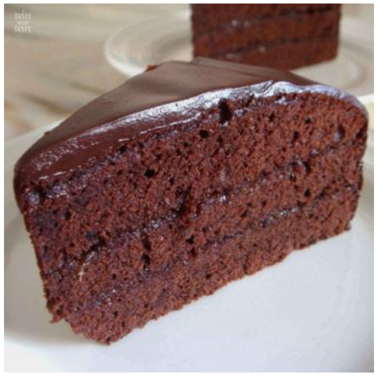	**4.26** 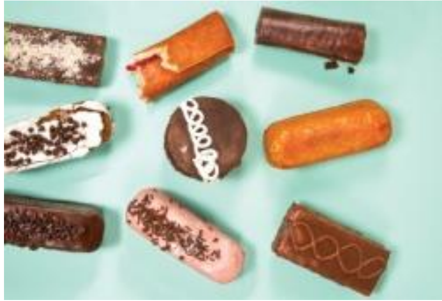	**4.27** 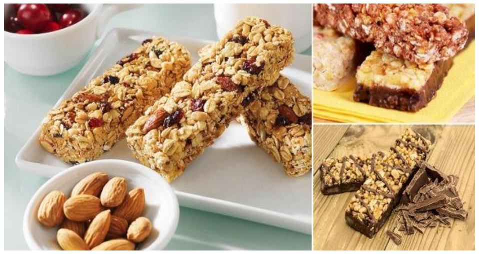

5.1

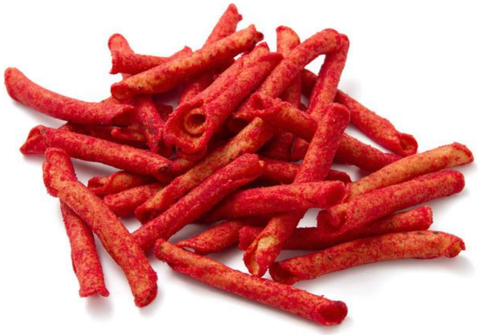

5.2

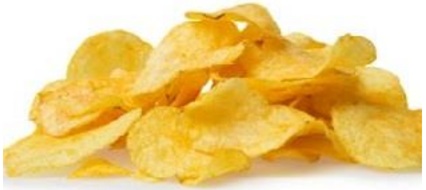

5.3

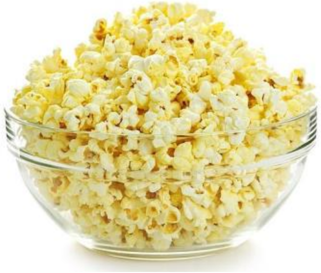

5.4

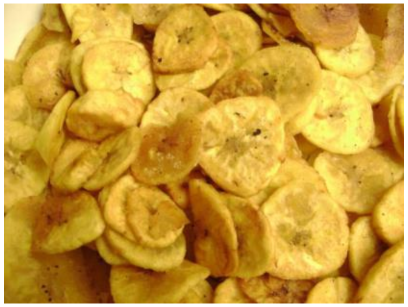

6.1

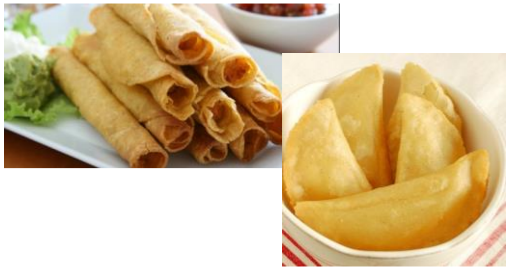

6.2

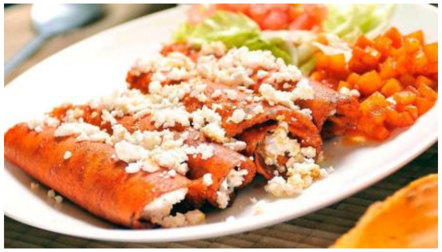

6.3

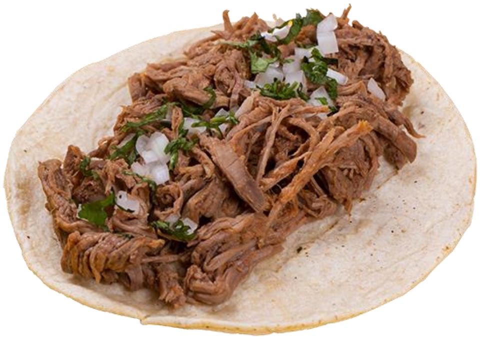

6.4

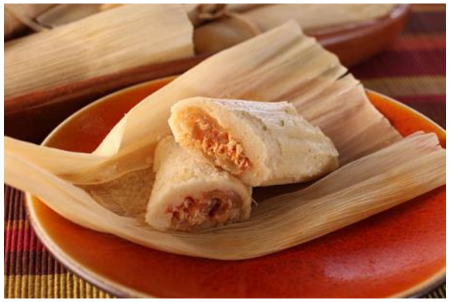

6.5

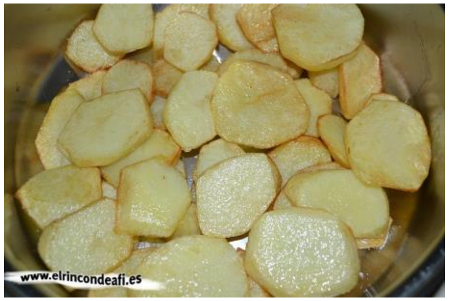

**6.6** 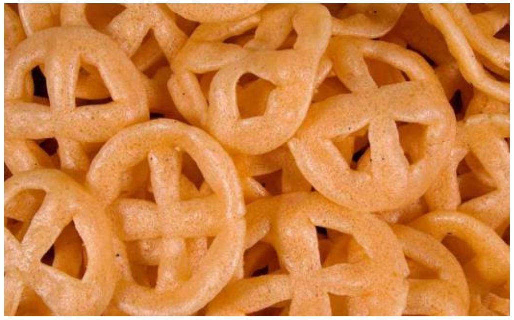	**6.7** 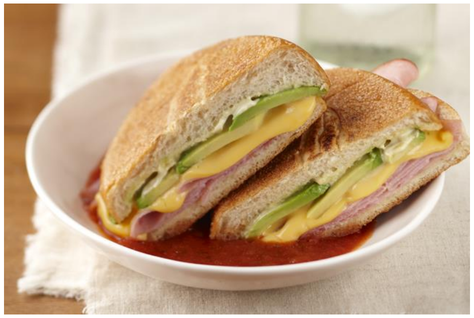	**6.8** 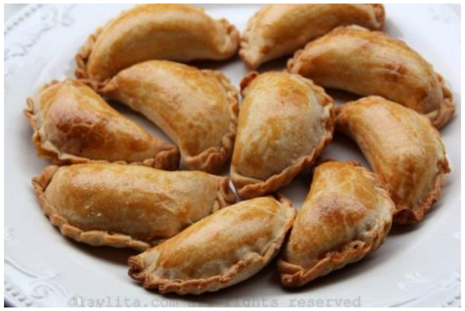
**6.9** 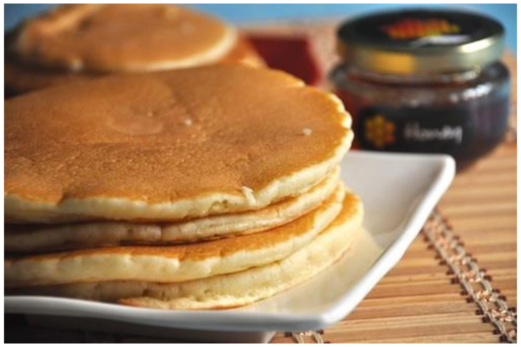	**6.10** 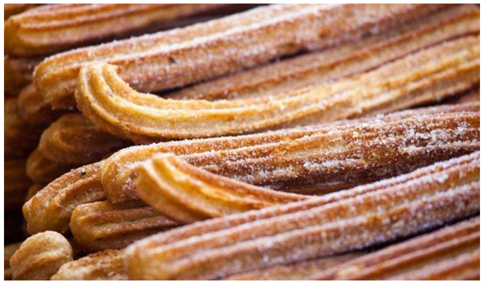	**7.1** 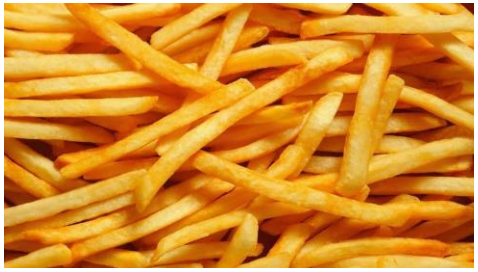
**7.2** 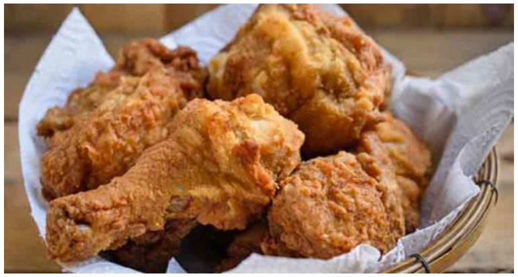	**7.3** 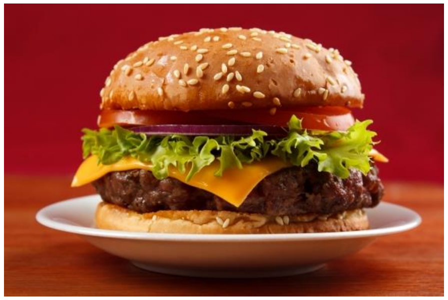	**7.4** 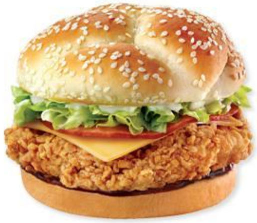

**7.5** 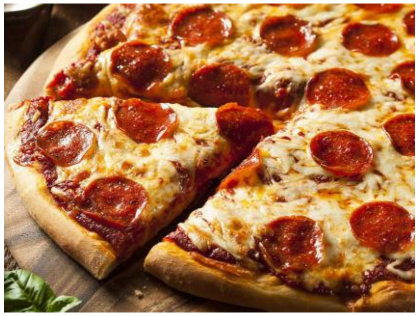	**7.6** 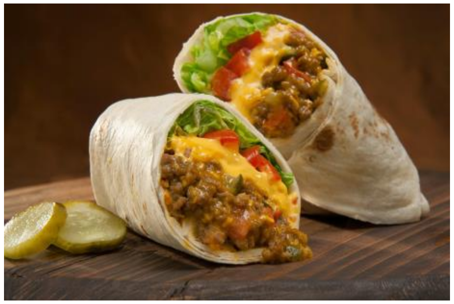	**7.7** 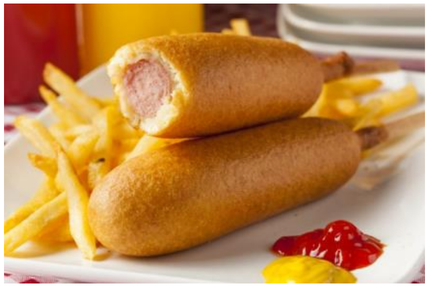
**8.1** 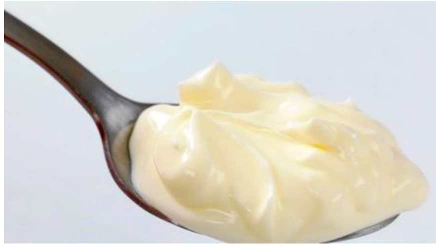	**8.2 y 8.3** 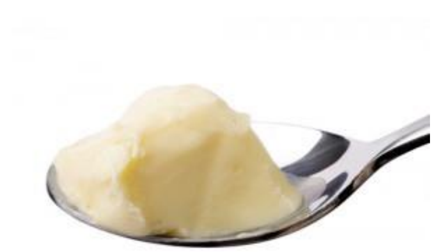	**8.4** 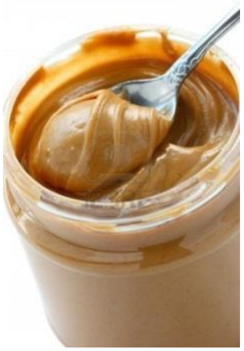
**8.5** 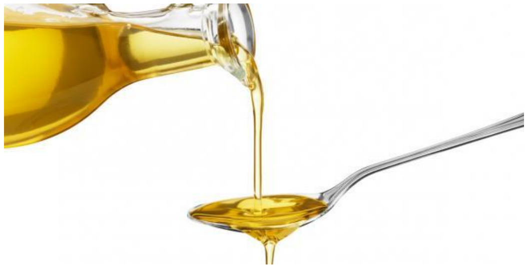	**8.6** 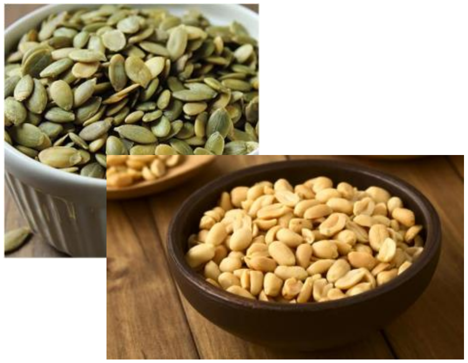	**8.7** 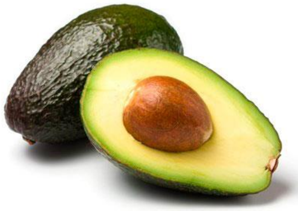

9.1

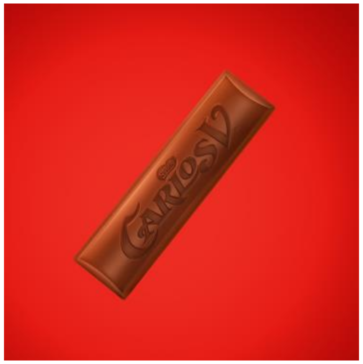

9.2

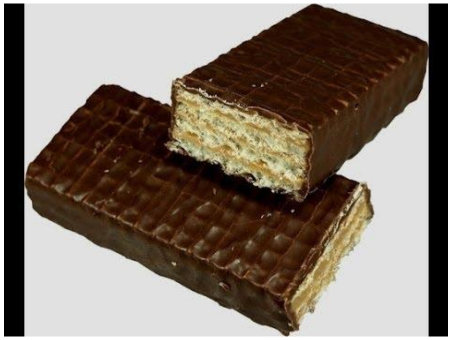

9.3

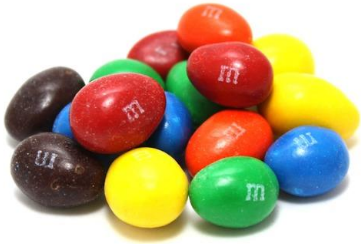

9.4

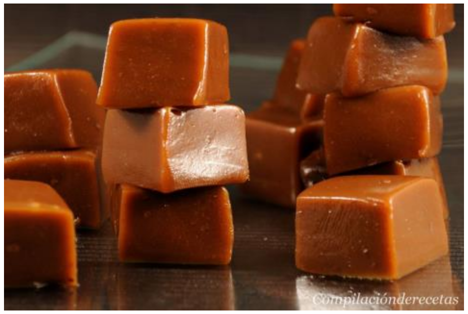

9.5

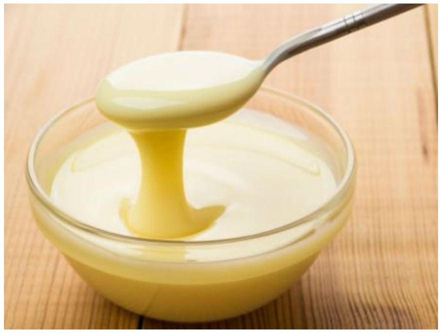

10.1

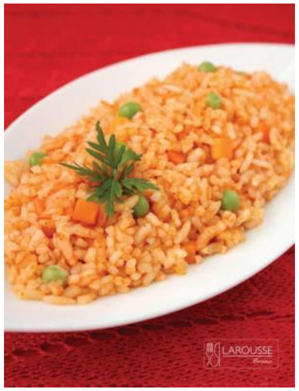

10.2

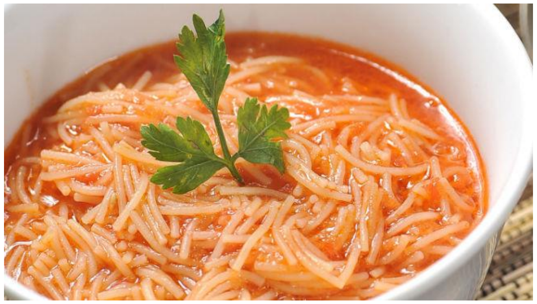

10.3

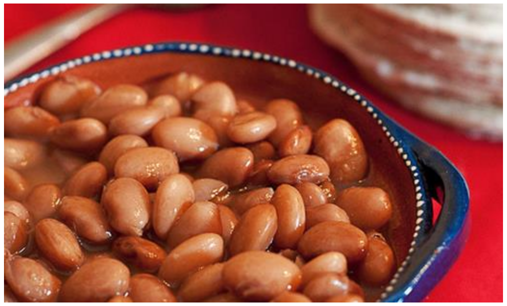

10.4

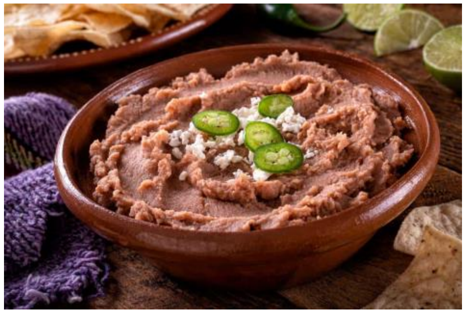

**10.5** 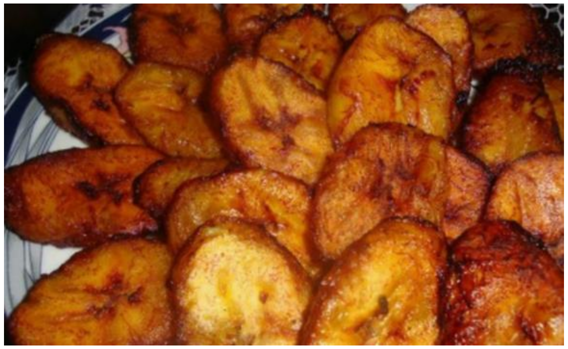	**10.6** 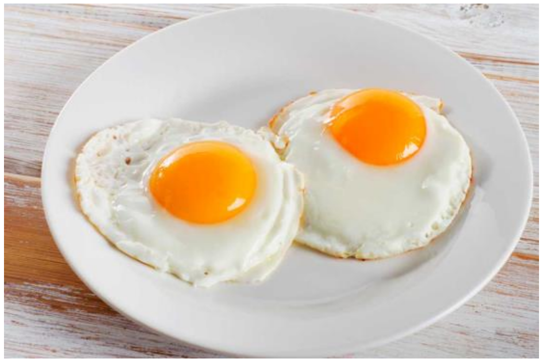	**10.7** 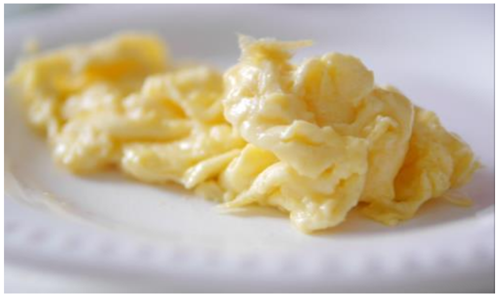
**10.8** 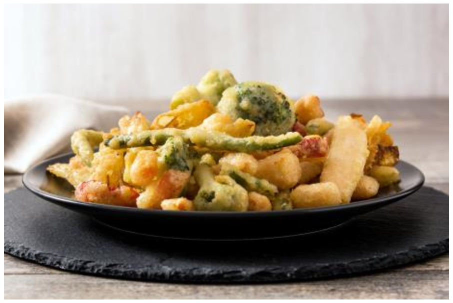	**10.9** 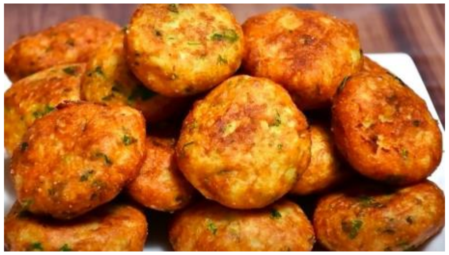	**10.10** 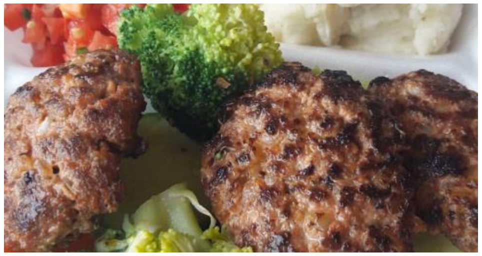
**10.11** 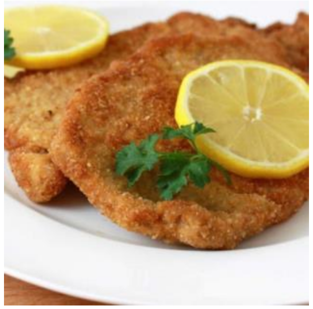	**10.12** 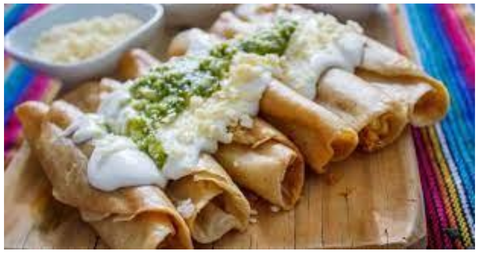	
